# Serum 25-hydroxyvitamin D and cancer-related fatigue: associations and effects on depression, anxiety, functional capacity and health-related quality of Life in breast cancer survivors during adjuvant endocrine therapy

**DOI:** 10.1186/s12885-022-09962-x

**Published:** 2022-08-06

**Authors:** Isis Danyelle Dias Custódio, Fernanda Silva Mazzutti Nunes, Mariana Tavares Miranda Lima, Kamila Pires de Carvalho, Débora Santana Alves, Juliana Freitas Chiaretto, Paula Philbert Lajolo Canto, Carlos Eduardo Paiva, Yara Cristina de Paiva Maia

**Affiliations:** 1 Molecular Biology and Nutrition Research Group, School of Medicine, Federal University of Uberlandia, Uberlandia, Minas Gerais, 38405-320 Brazil; 2 Department of Clinical Oncology, Clinic’s Hospital, Federal University of Uberlandia, Uberlandia, Minas Gerais, 38405-320 Brazil; 3 Department of Clinical Oncology, Barretos Cancer Hospital, Barretos, Sao Paulo, 14784-400 Brazil; 4 Nutrition Course, School of Medicine, Federal University of Uberlandia, Uberlandia, Minas Gerais, 38405-320 Brazil

**Keywords:** Fatigue, Vitamin D deficiency, Cancer survivors, Breast neoplasms, Aromatase Inhibitors, Health-related quality of life

## Abstract

**Background:**

The adjuvant treatment with Aromatase Inhibitor (AI) is considered standard of care for postmenopausal breast cancer (BC) women with hormone receptor-positive (HR +), however, it often causes adverse effects such as cancer-related fatigue (CRF). The high prevalence of vitamin D deficiency in postmenopausal women who start adjuvant AI supports the hypothesis that hypovitaminosis D would be one of the biological explanations for toxicity of AI. This study aimed to identify the relationship between 25-hydroxyvitamin D [25(OH)D] and CRF, and to analyze their associations and effects on depression, anxiety, functional disability, muscle/joint aches and HRQL.

**Methods:**

This prospective study included 89 postmenopausal women diagnosed with HR + early BC in adjuvant endocrine therapy with AI. Anthropometric and body composition assessments were performed, as well as dietary assessments by application of 24-h dietary recall, at three time points, totaling 24 months of follow-up. The women completed the Cervantes Scale (CS), Hospital Anxiety and Depression Scale (HADS) and Health Assessment Questionnaire (HAQ). The CRF was determined from the Functional Assessment of Chronic Illness Therapy-fatigue (FACIT-F). The serum 25(OH)D was determined by electrochemiluminescence, with cut-off point above 75 nmol/L adopted as sufficiency. Generalized Linear Model (GLzM) and Generalized Mixed Model (GMM) analysis were used.

**Results:**

At baseline, 36% (*n* = 32) of the women presented CRF and 39.3% (*n* = 35) had 25(OH)D below 75 nmol/L. None of the women reached the Estimated Average Requirements (EAR) of vitamin D. The causality between 25(OH)D and CRF was not significant. Longitudinally, lower levels of 25(OH)D had a negative effect on anxiety (*p* = 0.020), Menopause and Health (*p* = 0.033) and Vasomotor scores (*p* = 0.007). Also, the CRF had a negative effect on anxiety (*p* = 0.028); depression (*p* = 0.027); functional disability (*p* = 0.022); HRQL (*p* = 0.007); Menopause and Health (*p* = 0.042), Psychological (*p* = 0.008) and Couple Relations (*p* = 0.008) domains; and on Health (*p* = 0.019) and Aging (*p* = 0.036) subdomains. Vasomotor subdomain (β = -2.279, *p* = 0.045) and muscle/joint aches (β = -0.779, *p* = 0.013) were significant with CRF only at baseline.

**Conclusions:**

This study found negative effect of body adiposity on CRF. Still, the clinical relevance of 25(OH)D and CRF is highlighted, especially that of CRF, considering the consistent impact on several adverse effects reported by BC survivors during adjuvant endocrine therapy.

**Supplementary Information:**

The online version contains supplementary material available at 10.1186/s12885-022-09962-x.

## Background

Recently, female breast cancer (BC) has become the leading cause of cancer incidence worldwide [1]. In 2020, more than 2.2 million new cases of BC were estimated worldwide, and this number is expected to increase by more than 40% by 2040 [1].

The Aromatase Inhibitors (AI) are one of the adjuvant treatment options for postmenopausal BC women with hormone receptor-positive (HR +) [2]. Their mechanism of action is the inhibition or inactivation of aromatase, significantly reducing the plasma levels of estrogen from its androgenic precursors [2]. However, estrogen is involved in numerous physiological processes and, although related to the proliferation of tumor cells in HR + BC, it is expected that the depletion of this hormone generates significant adverse effects [3]. In this sense, the use of AI has been associated with negative effects on the urogenital system, interfering with sexual functioning [4]; depression [5]; increased risk of fractures and osteoporosis [6], joint pain or stiffness and fatigue [7].

Vitamin D deficiency has also been associated with symptoms such as non-specific joint pain, chronic fatigue and depression [5]. A 25-hydroxyvitamin D [25(OH)D] level of 75 nmol/L or higher has been associated with improved muscle strength and muscle pain syndrome, decreased risk of falls and fractures, reduced cytokine synthesis and lymphocytic proliferation [5], better tooth attachment [5, 8], improved depression and wellbeing [8, 9], reduction in the risk of autoimmune diseases, type 2 diabetes [5, 10], cardiovascular diseases [5, 10, 11], infectious [10] and neoplastic [5, 8, 10]. Estrogen has a positive effect on the activity of the vitamin D receptor and 1-alpha hydroxylase, an enzyme that converts 25(OH)D into biologically active 1,25-dihydroxyvitamin D [12]. Due to this, it is believed that the reduction of this hormone could unmask a 25(OH)D subclinical deficiency [12], which could intensify adverse effects related to the use of AI.

Furthermore, the vitamin D is involved in the modulating several inflammatory and pain pathways [13], in neurological [14] and oxidative [15] processes, in addition to calcium homeostasis [13], among others, which makes it essential for overall health [13], which is why its deficiency is one of the possible biological justifications for toxicity of AI.

The multicenter, prospective, and randomized trial (*n* = 500) identified that 41.2% (*n* = 206) of women with early-stage BC prematurely discontinued the hormone therapy with AI, with 79.1% (*n* = 163) due to the adverse effects. Of this percentage, the two main causes were musculoskeletal symptoms (73.6%, *n* = 120) and fatigue or insomnia (11.0%, *n* = 18) [16]. In addition to affecting treatment adherence, the adverse effects of adjuvant endocrine therapy greatly impact the health-related quality of life (HRQL) [3].

According to the National Comprehensive Cancer Network (NCCN), cancer-related fatigue (CRF) is “a distressing, persistent, subjective sense of physical, emotional and/or cognitive tiredness or exhaustion related to cancer or cancer treatment that is not proportional to recent activity and interferes with usual functioning” [17]. CRF causes many physical, psychosocial, and economic consequences [18], thus being a strong predictor of HRQL in BC survivors, even after treatment [17, 19]. CRF is often linked to reports of other symptoms such as pain, insomnia, cognitive dysfunction [19], depression and anxiety [19, 20].

There are several interventions that aim to reduce CRF, such as physical exercise [21], acupuncture [22], yoga [23], mindfulness-based interventions [24], psychological intervention [25], cognitive behavioral therapy [26], educational intervention [27], and vitamin D supplementation [28], among others, each with its own evidence and specific indication, considering the complex and multidimensional nature of this clinical condition [29].

Considering the above, the aim of this study was to identify the relationship between 25(OH)D and CRF, as well as to analyze their associations and effects on depression, anxiety, functional disability, muscle/joint aches, and HRQL in BC survivors during adjuvant endocrine therapy. We hypothesized that those women with lower 25(OH)D concentration and CRF, would have higher scores for both anxiety and depression, higher functional disability, worse HQRL and higher reports of muscle/joint aches. Also, we hypothesized that women with a worse nutritional status would have a worse score for CRF. To the best of our knowledge, this is the first prospective study to assess both the association and the effect of vitamin D and CRF on several aspects related to the health of BC survivors using AI.

## Methods

### Ethics statement, Study design and eligibility criteria

The study was approved by the Human Research Ethics Committee (nº. 1.331.949/15, addendum nº. 2.905.835/18) and conducted in accordance with the Declaration of Helsinki. A written free and informed consent was obtained from all participants.

From January 2016 to August 2018, postmenopausal women diagnosed with HR + early BC in adjuvant endocrine therapy with AI were consecutively recruited through the convenience non-probability sampling.

This prospective study was carried out at the Clinical Hospital of the Federal University of Uberlandia, Minas Gerais, Brazil. The face-a-face assessments were performed by properly trained researchers, at three time points: T0, baseline; T1, intermediate follow-up period, 12 months after T0; and T2, final follow-up period, 24 months after T0, totaling 24 months of follow-up.

The volunteers were included at any stage of the AI treatment, considering the following inclusion criteria: women aged between 18 and 80 years, who were HR + early BC and who had the physical, verbal and cognitive ability needed to respond to the tools necessary for the study. Eligible participants were excluded if they had metastasis, recurrence or contralateral BC, previous history of other cancers, another cancer concomitant with BC, age ≥ 80 years, wheelchair or bedridden, admission to palliative care and inability to attend collection. The diagram reporting the number of women recruited and selected in this study can be seen in the publication of Mazzutti and colleagues [30].

The collection of clinical and sociodemographic data occurred through the analysis of medical records or interviews.

### Anthropometric and Body composition measurement

The weight and height were obtained by a mechanical scale with 100 g sensitivity and a vertical stadiometer with a 1 mm precision scale, respectively. Regarding the waist circumference (WC) and hip circumference, a flexible and inelastic tape was used. All measures were carried according to the specific protocol [31].

To assess the risk of metabolic complications, we adopted the cut-off ≥ 80 cm for WC and > 0.85 for the waist-to-hip ratio (WHR) [32]. Additionally, to assess abdominal fat, we calculated the waist-to-height ratio (WHtR), which cut-off is ≥ 0.5 as indicator of excess abdominal fat [33], and the conicity index, which estimation consider weight, height and WC [34].

The body mass index (BMI) was calculated in Kg/m^2^ and the overweight was classified according to the age group: for the adult (age range 18—60 years), cut-off ≥ 25 kg/m^2^ [32]; and elderly population (≥ 60 years), cut-off > 27 kg/m^2^ [35].

The body composition was evaluated with horizontal tetra polar bioelectrical impedance analysis (BIA) (Biodynamics, model 450) according to the protocol [36, 37]. Considering the sensitivity of the exam to the presence of water body, we followed the recommended standardization of the method and the participants received pre-test guidelines in order to minimize measurement errors [36]. The body fat (BF) (in kilograms) was calculated by subtracting the fat free mass obtained using the predictive equation proposed by Kyle and collaborators [38] from the body weight and the percentage was obtained in relation to total body weight. The women whose exam detected water retention (total body water over 75%) were excluded from the BF analyses.

### Dietary data

At each time point (T0, T1 and T2), three nonconsecutive 24-h dietary recall (24HR) were applied by nutritionists, totaling nine 24HR per participant. The 24HR, one referring to a weekend, were applied face-to-face (the first) and through telephone interviews, according to the methodology used in the Vigitel Study [39].

The quantification of nutrients from the 24HR was estimated through the Nutrition Data System for Research (NDSR) software, version 2010 (Minneapolis, MN, USA). The following nutrients were evaluated regarding the 25(OH)D concentration: vitamin D, calcium, total fat, total monounsaturated fatty acids (MUFA), total polyunsaturated fatty acids (PUFA), omega-3, omega-6, magnesium, zinc and fiber. The dietary intake of vitamin D was also evaluated in relation to Estimated Average Requirements (EAR). Furthermore, consumption of milk product, fish and seafood, and egg, in grams, were analyzed considering their relationship with 25(OH)D level.

Due to intra- and inter-individual variability of food consumption, the data were deattenuated [40] using the PC-Side software (Department of Statistics, Iowa State University, Ames, IA, USA), and were adjusted by residual method by the mean energy of the sample [41].

### 25-hydroxyvitamin D

Venous blood collection was performed at the hospital on a pre-scheduled date.

The serum 25(OH)D concentration was measured in nanomoles per liter (nmol/L) using electrochemiluminescence. The survivors were dichotomized into two subgroups using the cut-off points based on the guidelines from the Brazilian Society of Endocrinology and Metabolism (SBEM) and the Brazilian Society of Clinical Pathology/Laboratory Medicine (SBPC), with value equal to or greater ( ≥) at 75 nmol/L (equivalent to 30 ng/mL) being considered sufficiency [42].

### Patient-reported outcome (PRO) instruments

All participants replied by interview to the 31-item Cervantes Scale (CS-31), Functional Assessment of Chronic Illness Therapy-fatigue (FACIT-F), Hospital Anxiety and Depression Scale (HADS) and Health Assessment Questionnaire (HAQ).

*CS-31.* This is a HRQL questionnaire that considers particularities of the perimenopausal and postmenopausal women, having been developed in 2004 [43] and validated in Brazil in 2012 [44]. The CS-31 consists of 31 items rated on a Likert scale from 0 to 5 and divided into four domains, namely Menopause and Health (subdivided into Vasomotor Symptoms, Health and Aging), Sexuality, Couple Relations and Psychological, with scores range from 0 to 155 points. In cases of one or two unanswered questions, we used correction factors, but questionnaires with three or more unanswered questions were considered invalid [43]. In this study, the Cronbach’s alpha were Global Score α = 0.89, Menopause and Health α = 0.81, Psychological α = 0.85, Sexuality α = 0.84, Couple Relations α = 0.75, Vasomotor Symptoms subdomain α = 0.80, Health subdomain α = 0.67, and Aging subdomain α = 0.67.

*FACIT-F* (version 4). This instrument of 40-item, validated in Brazil in 2010 [45], includes the 27-item Functional Assessment of Cancer Therapy-General (FACT-G) that assess the HRQL and 13 items that assess self-reported fatigue [46]. This scale measures four well-being subscales (physical, social/family, emotional and functional), one fatigue subscale (FACIT-Fatigue, score range 0–52), and derives to calculate the FACIT-F Trial Outcome Index (TOI) (score range 0–108), the FACT-G total score (score range 0–108) and the FACIT-F total score (score range 0–160). Items are rated on a Likert scale from 0 (not at all) to 4 (very much), with a higher score representing a better HRQL. The FACIT-Fatigue has a cut-off to identify presence of fatigue, with cut-off < 34 indicating clinically relevant fatigue [47]. In the present study, the Cronbach’s alpha was FACIT-Fatigue α = 0.89.

*HADS*. This self-reported questionnaire was developed in 1983 [48] and validated in Brazil in 1995 [49]. This scale comprising two subscales with seven items each, denominated HADS-A and HAD-D, which assessed anxiety and depression, respectively. Items are rated using a 4-point Likert scale with scores of 0 (minimally present) to 3 (maximally present), with higher scores indicating greater distress. The scores range from 0 to 21 and the following cut-off were adopted for both subscales: < 8 for non-cases, ≥ 8 for doubtful cases and ≥ 11 for the identification of cases [48]. In this study, the Cronbach’s alpha were HADS-A α = 0.76 and HADS-D α = 0.80.

*HAQ.* This instrument assesses functional disability [50], consisting of 20 items that determine the capacity for various activities assessed in the week prior to the application of the questionnaire, such as dressing, getting up, walking, performing hygiene, reaching and holding objects, assessing movements of the upper and lower limbs and both simultaneously [51]. The items are subdivided into 8 categories and evaluated on a 4-point Likert scale, with scores range from 0 (“without difficulty”) to 3 (“can't do it”), with higher scores representing greater disability [52]. This questionnaire was validated in Brazil in 1990 [53]. In this study, the HAQ presented Cronbach’s alpha α = 0.88.

### Statistical analysis

The sample size was calculated considering a group of individuals and three measurements. Using the G*Power software, version 3.1 (Düsseldorf, Germany) [54], an F test was conducted using ANOVA repeated measures, based on an effect size f of 0.25, an alpha level of 0.05 and at 80% power, 28 women were required women at each study time. For cross-sectional analyses, the sample was 89 women, while for prospective analyses, the 38 women who participated at three time points of study were considered. All participants were coded by numbers at data collection and, remained this way in the database.

The sample was stratified by demographic and clinical characteristics.

Factors that interfere with 25(OH)D concentration were evaluated according to the established cut-off. For these analyses, we used Chi-Square Independence Test, Fisher Exact Test, Test-t Independent and Mann–Whitney.

We used Cronbach’s alpha coefficient to assess the internal consistency of PRO Instruments, considering adequate values between 0.70 and 0.95 [55].

Generalized Linear Model (GLzM) and Generalized Mixed Model (GMM) analysis were used to verify, respectively, the associations and the effects (include effect of the time points and the interaction with the time points) of 25(OH)D concentration and FACIT-Fatigue score (independent variables) on the PRO Instruments HADS-A, HADS-D, HAQ, CS-31 Global score, domains and subdomains, and on the CS-31 item – “Aching in muscle and/or joints” (dependent variables). Furthermore, GLzM and GMM were used to investigate causality in the association between 25(OH)D concentration and CRF.

The causality in the association between 25(OH)D concentration and FACIT-Fatigue score was also analyzed with Spearman’s bivariate correlation. Correlation coefficients < 0.4 were considered weak correlations, between 0.4 and 0.6, moderate correlations, and > 0.6, strong correlations [56].

In addition, GLzM and GMM were used to verify the impact of anthropometric and body composition measurement (independent variables) on FACIT-Fatigue score (dependent variable).

All GLzM and GMM analysis included adjustment variables, as described in the respective tables. Regarding GMM, based on lowest Akaike Information Criterion (AIC) value, the best combination of the covariance matrices was AR1 (fixed effects) and variance components or AR1 (random effects). The adjustment method for multiple comparisons was Sidak.

We assessed the change in FACIT-Fatigue score between the three time points (T0, T1 and T2) considering a Minimum Clinically Important Difference (MCID) of 5% to classify the women between T0T1, T1T2, T0T2 and T0T1T2 into five clusters of CRF: The same, patients who maintained the FACIT-Fatigue score between T0T1, T1T2, T0T2 or who maintained the score at all three times (T0T1T2); Better, patients who improved the FACIT-Fatigue score between T0T1, T1T2, T0T2 or who improved at T1 and again at T2 (T0T1T2); Worse, patients who worsened the FACIT-Fatigue score between T0T1, T1T2, T0T2 or who worsened at T1 and again at T2 (T0T1T2); V, Patients who worsened the FACIT-Fatigue score at T1 and improved at T2 (T0T1T2); Inverted V, patients who improved the FACIT-Fatigue score at T1 and worsened at T2 (T0T1T2). The clusters in T0T1T2 were based on the classifications of each patient at T0T1 and again at T1T2.

The statistical analyzes were performed using IBM SPSS Statistics (Armonk, NY, USA), software package (SPSS Statistics for Windows, version 21.0), considering *p*-values < 0.05 statistically significant.

## Results

In the present study, we evaluated the medical records of 256 patients using AI and 107 patients were excluded from the selection for the following reasons: metastasis (*n* = 35), recurrence or contralateral BC (*n* = 20), age ≥ 80 years (*n* = 23), wheelchair or bedridden (*n* = 5), admission to palliative care (*n* = 6), previous history of other cancers (n = 5), another cancer concomitant with BC (n = 3), Alzheimer’s disease (*n* = 3), replacement with AI to tamoxifen (*n* = 2), withdrawal from treatment (*n* = 1), pulmonary edema (*n* = 1), pulmonary hypertension (*n* = 1), death (*n* = 1) and *Sjoergren’s* syndrome (*n* = 1). The eligibility assessment of 149 patients resulted in 56 exclusions: refusal to participate (*n* = 22), impossibility of telephone contact (*n* = 21), not under treatment ≥ 6 months (*n* = 12) and death (*n* = 1). Four patients were excluded from the study due to recurrence of BC (*n* = 1), incomplete questionnaires (*n* = 1) and non-attendance of all appointments (*n* = 2), totaling 89 women in the baseline (T0). For the prospective analyses, we considered the 38 women that participated at the three time points of the study, with the others having been excluded for the following causes: refusal to participate in the research (*n* = 16), impossibility of telephone contact (*n* = 13), end of treatment (*n* = 15), inability to attend collection (problems with commuting) (*n* = 6) and recurrence of BC (*n* = 1).

The demographic and clinical characteristics of the 89 BC survivors were analyzed (Table 1).

**Table 1:** Demographic and clinical characteristics of the breast cancer survivors during endocrine therapy

Characteristics	Overall (*n* = 89) n (%)
**Age (years) – median (p25-p75)**	65 (58.5–69.5)
< 60	25 (28.1)
≥ 60	64 (71.9)
**Marital Status**	
Single/ Divorced/Separated/Widow	50 (56.2)
Married	39 (43.8)
**Partner**	
No	22 (24.7)
Yes	67 (75.3)
**Educational Level**	
Below high school	61 (68.5)
High school or higher education	28 (31.5)
**Income (minimum wage)**	
< 3	53 (59.6)
≥ 3	36 (40.4)
**Work activity**	
Active	22 (24.7)
Inactive	67 (75.3)
**Surgery**	
Breast-conserving surgery	51 (57.3)
Mastectomy	38 (42.7)
**Prior Radiotherapy**	
No	14 (15.7)
Yes	75 (84.3)
**Prior Chemotherapy**	
No	21 (23.6)
Yes	68 (76.4)
**Chemotherapy Regimen**	
Adjuvant	53 (77.9)
Neoadjuvant	15 (22.1)
**Prior Tamoxifen**	
No	49 (55.1)
Yes	40 (44.9)
**Tumoral Subtype**	
Ductal	86 (96.6)
Lobular	3 (3.4)
**Clinical Stage**	
I	26 (29.2)
II	48 (53.9)
III	13 (14.6)
NR	2 (2.2)
**Tumor Grade**	
G1	14 (15.7)
G2	66 (74.2)
G3	5 (5.6)
NR	4 (4.5)
**Molecular Subtype**	
ER + and/or PR + , HER2- and Ki-67 < 14%	17 (19.1)
ER + and/or PR + , HER2- and Ki-67 ≥ 14%	37 (41.6)
ER + and/or PR + , HER2 +	29 (32.6)
NR	6 (6.7)
**Months since start on AI**	29.5 (18.1–41.8)
**Years since diagnosis**	4 (2—5)
**Years since last menstrual period**	16 (8—20)

Continuous variables are shown as median (p25-p75), and categorical variables are shown as absolute numbers and percentage frequency (in parentheses); Time point: T0, Baseline; Prior, before starting AI use; AI, aromatase inhibitor; *ER* estrogen receptor, *PR* progesterone receptor, *HER2* human epidermal growth factor type 2 receptor, *Ki 67* Ki 67 antigen, -, negative; + , positive; *NR* Not reported, *G1* Well-differentiated tumor (low grade), *G2* Moderately differentiated tumor (intermediate grade), *G3* Poorly differentiated tumor (high grade). The Brazilian minimum wage was R$ 880.00

Considering the overall, the medians (p25-p75) were 65 (58.5–69.5) years of age, 29.5 (18.1–41.8) months of time using AI, 4 (2–5) years of time diagnosis, and 16 (8–20) years of climacteric period. Regarding adjuvant endocrine therapy, 44.9% (*n* = 40) of women used tamoxifen prior to starting AI (Table 1). At baseline, 36.0% (*n* = 32) of women presented CRF and 39.3% (*n* = 35) had serum 25(OH)D levels below 75 nmol/L (Data no shown).

Considering the FACIT-Fatigue subgroups, we identified that those women with CRF had a lower median of time using AI (23.3 months) even when compared to the subgroup without CRF (33.6 months) (*p* = 0.028) (see Supplementary Table 1).

Among the factors that interfere with 25(OH)D concentration, we identified a significant difference regarding the season of the blood draw. In winter, the frequency of women with 25(OH)D concentration < 75 nmol/L was higher in relation to those with concentration ≥ 75 nmol/L (*p* = 0.039). In addition, those women with 25(OH)D concentration < 75 nmol/L had a lower median of MUFA/PUFA ratio (*p* = 0.012) and a higher median of omega-6 intake (*p* = 0.016) even when compared to the subgroup ≥ 75 nmol/L (Table 2).

**Table 2:** Factors that interfere with 25-hydroxyvitamin D concentration in the breast cancer survivors during endocrine therapy

Factors	Overall (*n* = 89)	25(OH)D		*p*
		** < 75 nmol/L(*n* = 35)**	** ≥ 75 nmol/L(*n* = 54)**	
**Age (years)**	64.0 (7.7)	64.6 (7.6)	63.6 (7.8)	0.470 ^*^
< 60	25 (28.1)	7 (28.0)	18 (72.0)	0.172^θ^
≥ 60	64 (71.9)	28 (43.8)	36 (56.3)	
**Educational Level**				
Below high school	61 (68.5)	26 (42.6)	35 (57.4)	0.347^θ^
High school or higher education	28 (31.5)	9 (32.1)	19 (67.9)	
**Race Group**				
White	83 (93.3)	33 (39.8)	50 (60.2)	1.000^℧^
Black	6 (6.7)	2 (33.3)	4 (66.7)	
**Income (minimum wage)**				
< 3	53 (59.6)	23 (43.4)	30 (56.6)	0.340^θ^
≥ 3	36 (40.4)	12 (33.3)	24 (66.7)	
**Physical exercise**				
No	53 (59.6)	22 (41.5)	31 (58.5)	0.262^θ^
Yes	36 (40.4)	13 (36.1)	23 (63.9)	
**Current Smoking**				
No	80 (89.9)	30 (37.5)	50 (62.5)	0.308^θ^
Yes	9 (10.1)	5 (55.6)	4 (44.4)	
**Alcohol Intake**				
No	66 (74.2)	29 (43.9)	37 (56.1)	0.131^θ^
Yes	23 (25.8)	6 (26.1)	17 (73.9)	
**Supplementation**				
No	75 (84.3)	29 (38.7)	46 (61.3)	0.193^℧^
Yes, vitamin D	3 (3.4)	0 (0.0)	3 (100.0)	
Yes, calcium	2 (2.2)	2 (100.0)	0 (0.0)	
Yes, both	9 (10.1)	4 (44.4)	5 (55.6)	
**Season of the blood draw**				
Summer	44 (49.4)	13 (29.5)	31 (70.5)	**0.039**^θ^
Autumn	30 (33.7)	12 (40.0)	18 (60.0)	
Winter	15 (16.9)	10 (66.7) ^a^	5 (33.3) ^b^	
**Months since start on AI**	29.5 (18.1–41.8)	25.5 (18.3 – 38.7)	30.0 (17.8 -48.1)	0.413 ^**^
**Daily Sun Exposure (minutes/day)**	30 (15—60)	30 (10—60)	30 (18.8—60)	0.313 ^**^
**BMI (Kg/m**^**2**^**)**	28.3 (25.4–31.4)	28.7 (25.4–31.4)	28.0 (25.3–31.6)	0.804 ^**^
**Body Fat (Kg)**	28.3 (24–34.9)	27.7 (24.8–36.2)	28.5 (23.9–34.3)	0.781 ^**^
**Body Fat (%)**	40.2 (36.8–44.4)	40.2 (36.7–47.6)	40.9 (37.6–44.3)	0.904 ^**^
**Calcium Concentration (mg/dL)**	9.4 (9.2–9.9)	9.4 (9.1–9.8)	9.5 (9.3–9.9)	0.173 ^**^
**Parathyroid Hormone Concentration (pg/mL)**	44.0 (33.2–55.3)	43.0 (32.6–57.5)	44.9 (34.2–54.3)	0.886 ^**^
**Milk product intake (g)**	112.1 (27.5–222.0)	123.3 (28.5–225.3)	96.04 (12.9–221.6)	0.491 ^**^
No	10 (11.2)	2 (20.0)	8 (80.0)	0.304 ^℧^
Yes	79 (88.8)	33 (41.8)	46 (58.2)	
**Fish and Seafood intake**				
No	72 (80.9)	31 (43.1)	41 (56.9)	0.174^θ^
Yes	17 (19.1)	4 (23.5)	13 (76.5)	
**Egg intake**				
No	55 (61.8)	20 (36.4)	35 (63.6)	0.467^θ^
Yes	34 (38.2)	15 (44.1)	19 (55.9)	
**Dietary Intake**				
**Vitamin D (µg)**	3.5 (2.3–5.0)	3.5 (2.4–5.0)	3.5 (2.2–5.0)	0.967 ^**^
**Calcium (mg)**	475.8 (375.1–586.7)	464.9 (377.5–558.8)	478.2 (371.1–591.9)	0.788 ^**^
**Total Fat (g)**	47.8 (5.0)	47.0 (4.3)	48.4 (5.4)	0.196 ^*^
**MUFA (g)**	16.4 (2.4)	15.8 (2.2)	16.8 (2.4)	0.478 ^*^
**PUFA (g)**	12.0 (1.7)	12.2 (1.5)	11.9 (1.8)	0.052 ^*^
**MUFA/PUFA Ratio**	1.4 (1.2–1.6)	1.3 (1.1–1.4)	1.4 (1.3–1.6)	0.012 ^**^
**Omega-3 (g)**	1.5 (1.4–1.6)	1.5 (1.4–1.6)	1.5 (1.4–1.6)	0.551 ^**^
**Omega-6 (g)**	10.4 (1.5)	10.7 (1.2) ^a^	10.2 (1.7) ^b^	**0.016** ^*^
**n6/n3 fatty acids ratio**	7.0 (6.5 – 7.4)	7.1 (6.9 – 7.4)	6.7 (6.3 – 7.5)	0.193 ^**^
**Magnesium (mg)**	192.6 (173.6–216.4)	195.8 (176.1–221.4)	192.6 (172.8–207.7)	0.518 ^**^
**Zinc (mg)**	8.7 (1.4)	8.8 (1.5)	8.6 (1.4)	0.534 ^*^
**Fiber (g)**	15.7 (3.8)	16.5 (4.1)	15.1 (3.5)	0.113 ^*^

Continuous variables are shown as mean (standard deviation) or median (p25-p75), and categorical variables are shown as absolute numbers and percentage frequency (in parentheses); Time point: T0, Baseline; 25(OH)D, 25-hydroxyvitamin D; AI, aromatase inhibitor; *BMI* Body Mass Index, *MUFA* Total Monounsaturated Fatty Acids, *PUFA* Total Polyunsaturated Fatty Acids, n6/n3 fatty acids ratio, estimated ratio of omega-6 to omega-3 fatty acids. The Brazilian minimum wage was R$ 880.00

^θ^Chi-Square Independence Test

^℧^Fisher Exact Test

^*^ Test-t Independent

^**^ Mann–Whitney. Different superscript letters represent statistical significance when comparing column proportions. Bold value is statistically significant at *p* < 0.05

The dietary intake of vitamin D did not differ significantly between the 25(OH)D levels subgroups (*p* = 0.967). However, it is important to note that none of the women reached the Estimated Average Requirements (EAR) of vitamin D (10 µg/day or 400 IU, [57]), at baseline or in the prospective phase. The mean and median (p25-p75) intake, 3.7 ± 1.7 µg/day and 3.5(2.3–5.0)µg/day, respectively, were low in the overall, including those women who used vitamin D supplementation (Table 2). Considering only the women who reported supplement use (*n* = 12), the mean and median (p25-p75) intake of this nutrient were 4.5 ± 1.2 µg/day and 4.7 (3.5–5.7)µg/day, respectively (Data no shown).

Regarding the baseline, no significant association were found between 25(OH)D concentration and PRO Instruments. However, considering the longitudinal phase, we identified that low 25(OH)D level had negative effect on anxiety (*p* = 0.020), Menopause and Health score (*p* = 0.033) and Vasomotor score (*p* = 0.007). The time of study had effect on anxiety (*p* = 0.018) indicating that the women started endocrine therapy with more symptoms of anxiety, with an improvement over time. Still, the time of study had effect on Sexuality score (p = 0.036), with worse score in T2 compared to T1 (Table 3).

**Table 3:** Associations and effects of 25(OH)D on PRO Instruments in the baseline and in the longitudinal phase

PRO Instrument	Baseline (*n* = 89)							Longitudinal phase^1^ (*n* = 38)						
	**25(OH)D**		**β**	**95% CI**		**Wald Chi-square**	*p	**Time points**		**25(OH)D**		**Model Effects Test**		
	** < 75 nmol/L**	** ≥ 75 nmol/L**		**Lower**	**Upper**					** < 75 nmol/L**	** ≥ 75 nmol/L**	**Effects**	**Df**	**p
**HADS-A**	7.95 ± 1.02	8.09 ± 0.97	-0.017	-0.244	0.211	0.021	0.886			8.34 ± 0.94^a^	6.69 ± 0.84^b^			
								**T0**	10.16 ± 1.20^a^	10.85 ± 1.35	9.48 ± 1.25	**Time points**	2	**0.018**
								**T1**	7.47 ± 0.91^b^	8.50 ± 1.18	6.45 ± 0.89	**25(OH)D**	1	**0.020**
								**T2**	4.90 ± 1.21^c^	5.67 ± 1.28	4.14 ± 1.31	**Interaction**^**2**^	2	0.822
**HADS-D**	6.32 ± 1.10	7.57 ± 1.02	-1.249	-3.170	0.672	1.623	0.203			7.12 ± 1.08	6.82 ± 0.97			
								**T0**	7.21 ± 1.36	7.05 ± 1.50	7.37 ± 1.42	**Time points**	2	0.950
								**T1**	6.88 ± 1.04	7.29 ± 1.38	6.47 ± 1.01	**25(OH)D**	1	0.692
								**T2**	6.81 ± 1.38	7.02 ± 1.45	6.61 ± 1.48	**Interaction**^**2**^	2	0.647
**HAQ**	0.96 ± 0.15	1.08 ± 0.14	-0.112	-0.374	0.150	0.699	0.403			0.92 ± 0.14	0.91 ± 0.12			
								**T0**	0.88 ± 0.17	0.90 ± 0.20	0.87 ± 0.18	**Time points**	2	0.693
								**T1**	0.95 ± 0.13	1.06 ± 0.17	0.84 ± 0.13	**25(OH)D**	1	0.946
								**T2**	0.91 ± 0.18	0.79 ± 0.19	1.02 ± 0.19	**Interaction**^**2**^	2	0.063
**CS-31 Total Score**	65.98 ± 6.85	74.20 ± 6.10	-8.219	-20.958	4.521	1.599	0.206			62.88 ± 8.25	54.62 ± 7.44			
								**T0**	61.89 ± 10.09	67.67 ± 11.32	56.11 ± 10.42	**Time points**	2	0.856
								**T1**	57.53 ± 7.83	59.87 ± 10.66	55.19 ± 7.69	**25(OH)D**	1	0.189
								**T2**	56.83 ± 10.41	61.10 ± 11.13	52.55 ± 11.26	**Interaction**^**2**^	2	0.800
**CS-31 Menopause and Health Domain**	36.26 ± 3.55	39.80 ± 3.15	-3.534	-10.126	3.059	1.104	0.293			32.88 ± 3.77^a^	26.47 ± 3.37^b^			
								**T0**	29.32 ± 4.61	31.53 ± 5.30	27.11 ± 4.73	**Time points**	2	0.747
								**T1**	30.72 ± 3.53	33.33 ± 4.78	28.12 ± 3.47	**25(OH)D**	1	**0.033**
								**T2**	28.99 ± 4.77	33.79 ± 5.17	24.19 ± 5.19	**Interaction**^**2**^	2	0.592
**CS-31 Psychological Domain**	13.78 ± 3.02	16.27 ± 2.69	-2.493	-8.113	3.127	0.756	0.385			11.78 ± 3.43	10.70 ± 2.95			
								**T0**	16.45 ± 4.07	17.44 ± 4.86	15.46 ± 4.14	**Time points**	2	0.217
								**T1**	10.62 ± 3.23	12.40 ± 4.82	8.85 ± 3.13	**25(OH)D**	1	0.715
								**T2**	6.64 ± 4.22	5.50 ± 4.69	7.79 ± 4.67	**Interaction**^**2**^	2	0.597
**CS-31 Sexuality Domain**	11.68 ± 1.48	13.20 ± 1.31	-1.520	-4.267	1.226	1.177	0.278			10.77 ± 1.89	13.02 ± 1.65			
								**T0**	10.91 ± 2.29^a,b^	10.42 ± 2.74	11.41 ± 2.32	**Time points**	2	**0.036**
								**T1**	10.02 ± 1.75^a^	7.79 ± 2.44	12.25 ± 1.72	**25(OH)D**	1	0.155
								**T2**	14.75 ± 2.38^b^	14.09 ± 2.65	15.40 ± 2.62	**Interaction**^**2**^	2	0.350
**CS-31 Couple Relations Domain**	4.50 ± 1.38	4.80 ± 1.23	-0.306	-2.868	2.256	0.055	0.815			5.12 ± 1.51	5.40 ± 1.31			
								**T0**	4.31 ± 1.81	5.42 ± 2.05	3.21 ± 1.87	**Time points**	2	0.750
								**T1**	5.30 ± 1.51	3.84 ± 2.38	6.77 ± 1.46	**25(OH)D**	1	0.831
								**T2**	6.17 ± 1.86	6.11 ± 2.00	6.22 ± 2.04	**Interaction**^**2**^	2	0.126
**CS-31 Vasomotor Subdomain**	9.62 ± 1.17	9.40 ± 1.04	0.216	-1.950	2.381	0.038	0.845			9.04 ± 1.30^a^	5.90 ± 1.12^b^			
								**T0**	7.07 ± 1.56	7.73 ± 1.75	6.41 ± 1.62	**Time points**	2	0.774
								**T1**	8.02 ± 1.34	9.80 ± 2.16	6.24 ± 1.28	**25(OH)D**	1	**0.007**
								**T2**	7.33 ± 1.60	9.60 ± 1.71	5.06 ± 1.75	**Interaction**^**2**^	2	0.108
**CS-31 Health Subdomain**	11.62 ± 1.34	13.01 ± 1.19	-1.393	-3.878	1.091	1.208	0.272			9.18 ± 1.79	8.05 ± 1.61			
								**T0**	9.46 ± 2.08	9.86 ± 2.46	9.05 ± 2.10	**Time points**	2	0.807
								**T1**	8.80 ± 1.57	8.95 ± 2.18	8.66 ± 1.56	**25(OH)D**	1	0.464
								**T2**	7.58 ± 2.22	8.74 ± 2.65	6.43 ± 2.53	**Interaction**^**2**^	2	0.831
**CS-31 Aging Subdomain**	15.02 ± 1.92	17.38 ± 1.71	-2.356	-5.921	1.208	1.679	0.195			14.24 ± 1.98	12.89 ± 1.78			
								**T0**	12.42 ± 2.43	12.88 ± 2.75	11.96 ± 2.50	**Time points**	2	0.804
								**T1**	13.61 ± 1.87	13.36 ± 2.51	13.85 ± 1.83	**25(OH)D**	1	0.372
								**T2**	14.66 ± 2.51	16.47 ± 2.70	12.84 ± 2.72	**Interaction**^**2**^	2	0.364
**CS-31 Aching in muscles and/or joints**	3.38 ± 0.34	3.90 ± 0.32	-0.514	-1.114	0.085	2.828	0.093			3.47 ± 0.38	2.99 ± 0.31			
								**T0**	3.15 ± 0.45	3.07 ± 0.52	3.24 ± 0.46	**Time points**	2	0.917
								**T1**	3.32 ± 0.38	3.81 ± 0.62	2.82 ± 0.36	**25(OH)D**	1	0.150
								**T2**	3.23 ± 0.45	3.54 ± 0.49	2.92 ± 0.50	**Interaction**^**2**^	2	0.126

^*^ Generalized linear models (GLzM) ^**^ General Mixed Model (GMM); Data adjusted for age, education level, income, usage time of aromatase inhibitors and body mass index. Time point: T0, Baseline; *T1* Intermediate period, corresponding to 12 months after T0; and T2, Final follow-up period, corresponding to 24 months after T0, *PRO Instrument* Patient-Reported Outcome Instrument, *25(OH)D* 25-hydroxyvitamin D, *HADS-A* Hospital Anxiety and Depression Scale, subscale anxiety; HADS-D: Hospital Anxiety and Depression Scale, subscale depression; *HAQ* Health Assessment Questionnaire, *CS-31* 31-item Cervantes Scale, *SD* Standard deviation, *Df* degrees of freedom. Significant Model Effects Test are in bold. Sidak test: Different superscript letters represent statistical significance when comparing pairs, *p* value < 0.05. ^1^Longitudinal phase: PRO Instruments are shown as mean ± SD; ^2^Interaction between FACIT-F TOI and time points of study

We investigated direct and reverse causality in the association between 25(OH)D concentration and CRF, but no significance was found in models effect tests. In longitudinal phase, the time of study had significant effect on 25(OH)D concentration, with lower level in T2 compared T1 (*p* = 0.045), but only in model 1 with data adjusted for age (see Supplementary Table 2).

The causality was also investigated by bivariate correlation analysis, but without significance (r = -0.071, 95% [CI] = -0.310–0.160, *p* = 0.509) (Data no shown).

**Fig. 1:**
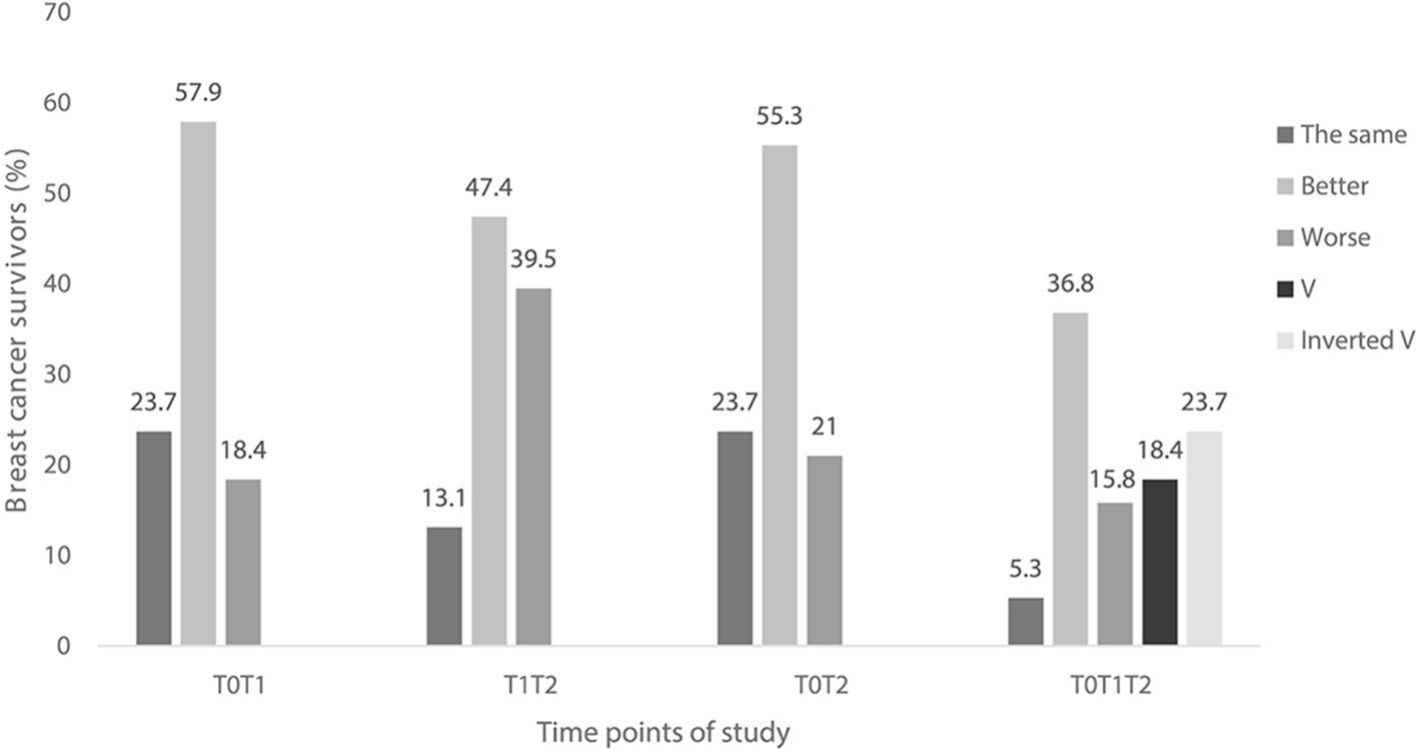
Distribution of breast cancer survivors in the FACIT-Fatigue clusters throughout the study (*n* = 38). Clusters developed from the 5% MCID between T0T1, T1T2, T0T2 and T0T1T2: The same, Patients who maintained the FACIT-Fatigue score between T0T1, T1T2, T0T2 or who maintained the score at all three times (T0T1T2); Better, Patients who improved the FACIT-Fatigue score between T0T1, T1T2, T0T2 or who improved at T1 and again at T2 (T0T1T2); Worse, Patients who worsened the FACIT-Fatigue score between T0T1, T1T2, T0T2 or who worsened at T1 and again at T2 (T0T1T2); V, Patients who worsened the FACIT-Fatigue score at T1 and improved at T2 (T0T1T2); Inverted V, Patients who improved the FACIT-Fatigue score at T1 and worsened at T2 (T0T1T2). Chi-square Independence Test showed that there was no association between time points of study and clusters, considering T0T1 and T1T2 [X2(2) = 4.452; *p* = 0.108]

Considering the clusters developed from the 5% MCID between the time points of study, we identified that the greater percentage of women was classified as “better”, i.e. with improvement in FACIT-Fatigue score between T0T1 (57.9%, *n* = 22), T1T2 (47.4%, *n* = 18), T0T2 (55.3%, *n* = 21) and T0T1T2 (36.8%, *n* = 14) (Fig. 1).

Considering the multiple causes that may be involved in the development and, or in increasing CRF, we identified that there was no statistically significant difference between FACIT-Fatigue subgroups regarding age, race, educational level, income and clinical stage (see Supplementary Table 1), and neither in relation to physical exercise (*p* = 0.980) (Data no shown). Still, the dietary intake of vitamin D did not differ significantly between the women with CRF (3.8 ± 1.8 µg/day) and without CRF (3.7 ± 1.7 µg/day) (*p* = 1.000) (Data no shown).

Moreover, we investigated the association of CRF with anthropometric and body composition parameters (Table 4), considering that these variables also may be related to CRF. At baseline, the women with lower FACIT-Fatigue score presented higher BMI (β = -0.637, CI = -0.986 to -0.287, *p* < 0.001), WC (β = -0265, CI = -0.427 to -0.103, *p* = 0.001), WHtR (β = -41.972, CI = -67.155 to -16.788, *p* = 0.001) and body fat (Kg) (β = -0.285, CI = -0.526 to -0.045, *p* = 0.020) (Table 4). In the longitudinal phase, the WC (*p* = 0.001) and conicity index (*p* = 0.021) had negative effect on CRF, and those women with a lower FACIT-Fatigue score presented WC > 80 cm and conicity index above the median (> 1.3) (Table 4). Considering the FACIT-Fatigue subgroups, we identified significant difference in relation the BMI (*p* = 0.002), WC (*p* = 0.004) and WHtR (*p* = 0.002), with women with CRF presenting worse scores (Table 4).

**Table 4:** Associations and effects of anthropometric and body composition parameters on cancer-related fatigue

Independent variable^1^	Baseline (*n* = 89)								Longitudinal phase (*n* = 38) FACIT-Fatigue (Mean ± SD)							
	**FACIT-Fatigue**		***p***	**β**	**95% CI**		**Wald Chi-Square**	*p	**Independent Variable**		**Time points**			**Model Effects Test**		
	**Score < 34 (*n* = 32)**	**Score ≥ 34 (*n* = 57)**			**Lower**	**Upper**					**T0**	**T1**	**T2**	**Effects**	**Df**	**p
**BMI (Kg/m**^**2**^**)**	30.5 (26.3–34.9)	26.8 (25.1–30.1)	**0.002**^**‡**^	-0.637	-0.986	-0.287	12.760	** < 0.001**	**BMI (Kg/m**^**2**^**)**		36.64 ± 2.75	39.01 ± 2.11	40.45 ± 2.86	**Time points**	2	0.547
									Without overweight	37.07 ± 2.68	36.58 ± 3.45	36.32 ± 2.86	38.31 ± 3.32	**BMI**	1	0.223
									Overweight	40.33 ± 2.20	36.69 ± 2.76	41.71 ± 2.27	42.59 ± 3.18	**Interaction**^**2**^	2	0.083
**WC (cm)**	98.0 (88.6–102.9)	90.5 (82.0–95.0)	**0.004**^**‡**^	-0.265	-0.427	-0.103	10.310	**0.001**	**WC (cm)**		40.94 ± 2.92	45.65 ± 2.87	42.30 ± 3.06	**Time points**	2	0.137
									< 80	46.98 ± 3.00^a^	44.80 ± 3.82	51.75 ± 4.61	44.40 ± 3.81	**WC**	1	**0.001**
									≥ 80	38.94 ± 2.04^b^	37.08 ± 2.67	39.55 ± 2.06	40.21 ± 2.91	**Interaction**^**2**^	2	0.243
**WHR**	0.91 (0.07)	0.9 (0.1)	0.214^**†**^	-19.649	-49.249	9.951	1.693	0.193	**WHR**		37.78 ± 2.70	40.27 ± 2.14	41.03 ± 2.91	**Time points**	2	0.513
									≤ 0.85	40.52 ± 2.32	38.44 ± 3.11	41.29 ± 2.68	41.82 ± 3.22	**WHR**	1	0.402
									> 0.85	38.87 ± 2.24	37.13 ± 2.82	39.25 ± 2.27	40.23 ± 3.18	**Interaction**^**2**^	2	0.964
**WHtR**	0.6 (0.6–0.7)	0.6 (0.5–0.6)	**0.002**^**‡**^	-41.972	-67.155	-16.788	10.670	**0.001**	**WHtR**		38.83 ± 3.50	44.29 ± 2.92	41.87 ± 3.63	**Time points**	2	0.196
									< 0.5	43.77 ± 3.95	39.91 ± 5.46	48.78 ± 4.77	42.61 ± 5.60	**WHtR**	1	0.249
									≥ 0.5	39.56 ± 2.03	37.75 ± 2.66	39.79 ± 2.06	41.13 ± 2.88	**Interaction**^**2**^	2	0.329
**Body Fat (Kg)**	31.5 (25.2–40.2)	27.7 (23.4–32.9)	0.092^**‡**^	-0.285	-0.526	-0.045	5.393	**0.020**	**Body Fat (Kg)**		38.79 ± 2.94	40.76 ± 2.39	39.59 ± 3.32	**Time points**	2	0.348
									≤ 28.21	40.04 ± 2.45	40.42 ± 3.27	40.04 ± 2.71	39.67 ± 3.34	**Body Fat (Kg)**	1	0.770
									> 28.21	39.39 ± 2.70	37.16 ± 3.25	41.48 ± 2.74	39.51 ± 3.94	**Interaction**^**2**^	2	0.259
**Body Fat (%)**	41.2 (36.9–47.8)	40.0 (36.8–43.7)	0.271^**‡**^	-0.322	-0.718	0.074	2.543	0.111	**Body Fat (%)**		38.61 ± 2.94	41.04 ± 2.38	40.53 ± 3.28	**Time points**	2	0.384
									≤ 40.35	39.56 ± 2.46	39.18 ± 3.24	40.26 ± 2.72	39.23 ± 3.33	**Body Fat (%)**	1	0.627
									> 40.35	40.56 ± 2.59	38.03 ± 3.23	41.82 ± 2.68	41.83 ± 3.83	**Interaction**^**2**^	2	0.539
**Conicity Index**	1.3 (0.1)	1.3 (0.1)	0.152^**†**^	-20.073	-45.098	4.953	2.471	0.116	**Conicity Index**		36.79 ± 2.73	39.54 ± 2.13	40.13 ± 2.95	**Time points**	2	0.409
									≤ 1.3	40.62 ± 2.18^a^	39.21 ± 2.84	40.49 ± 2.41	42.18 ± 3.08	**CI**	1	**0.021**
									> 1.3	37.01 ± 2.29^b^	34.36 ± 3.03	38.59 ± 2.34	38.08 ± 3.17	**Interaction**^**2**^	2	0.475

^**†**^Test-t Independent; ^**‡**^Mann–Whitney; Continuous variables are shown as mean (standard deviation) or median (p25-p75); *Generalized linear models (GLzM); **General Mixed Model (GMM); Data adjusted for age, education level, income and usage time of aromatase inhibitors. FACIT-Fatigue, Functional Assessment of Chronic Illness Therapy—Fatigue Scale, with cut-off < 34 indicating cancer-related fatigue; Time point: T0, Baseline; T1, Intermediate period, corresponding to 12 months after T0; and T2, Final follow-up period, corresponding to 24 months after T0; SD, Standard deviation; Df, degrees of freedom; BMI, body mass index; WC, waist circumference; WHR, waist-to-hip ratio; WHtR, waist-to-height ratio. Significant Model Effects Test are in bold. Sidak test: Different superscript letters represent statistical significance when comparing pairs, p value < 0.05. ^1^Continuous variable. ^2^Interaction between anthropometric/ body composition parameters and time points of study. The variables Body Fat and Conicity Index were categorized by the medians

At baseline, negative associations were observed between FACIT-Fatigue and PRO Instruments, indicating that the women with CRF presented more anxiety (β = -3.779, CI = -5.498 to -2.059, *p* < 0.001), depression (β = -4.799, CI = -6.559 to -3.038, *p* < 0.001), functional disability (β = -0.554, CI = -0.803 to -0.304, *p* < 0.001) and muscle/joint aches (β = -0.779, CI = -1.394 to -0.165, *p* = 0.013). Also, these women presented worse HRQL (β = -34.337, CI = -45.278 to -23.397, *p* < 0.001) and worse score in the following domains and subdomains of the CS-31: Menopause and health (β = -17.143, CI = -22.882 to -11.405, *p* < 0.001), Psychological (β = -16.214, CI = -20.792 to -11.636, *p* < 0.001), Vasomotor (β = -2.279, CI = -4.509 to -0.050, *p* = 0.045), Health (β = -6.325, CI = -8.513 to -4.138, *p* < 0.001) and Aging (β = -8.539, CI = -11.777 to -5.301, *p* < 0.001) (Table 5).

**Table 5:** Associations and effects of cancer-related fatigue on PRO instruments

PRO Instrument	Baseline (*n* = 89)							Longitudinal phase^1^ (*n* = 38)						
	**FACIT-Fatigue**		**β**	**95% CI**		**Wald Chi-square**	*p	**Time points**		**FACIT-Fatigue**		**Model Effects Test**		
	**Score < 34**	**Score ≥ 34**		**Lower**	**Upper**					**Score < 34**	**Score ≥ 34**	**Effects**	**Df**	**p
**HADS-A**	10.94 ± 0.98^a^	7.16 ± 0.86^b^	-3.779	-5.498	-2.059	18.546	** < 0.001**			8.21 ± 1.11^a^	6.57 ± 0.89^b^			
								**T0**	9.33 ± 1.15^a^	10.43 ± 1.25	8.22 ± 1.17^a^	**Time points**	2	**0.035**
								**T1**	6.91 ± 0.96^b^	7.56 ± 1.22	6.26 ± 0.90^b^	**FACIT-Fatigue**	1	**0.028**
								**T2**	5.94 ± 1.38^a,b^	6.63 ± 1.72	5.25 ± 1.31^a,b^	**Interaction**^**2**^	2	0.602
**HADS-D**	10.15 ± 1.00^a^	5.35 ± 0.88^b^	-4.799	-6.559	-3.038	28.544	** < 0.001**			8.22 ± 1.19^a^	6.61 ± 0.98^b^			
								**T0**	7.50 ± 1.29	8.59 ± 1.40	6.42 ± 1.32	**Time points**	2	0.697
								**T1**	7.15 ± 1.06	7.79 ± 1.31	6.50 ± 1.00	**FACIT-Fatigue**	1	**0.027**
								**T2**	7.60 ± 1.47	8.30 ± 1.72	6.91 ± 1.41	**Interaction**^**2**^	2	0.676
**HAQ**	1.39 ± 0.14^a^	0.83 ± 0.12^b^	-0.554	-0.803	-0.304	18.947	** < 0.001**			1.02 ± 0.16^a^	0.76 ± 0.13^b^			
								**T0**	0.81 ± 0.17	0.95 ± 0.19	0.68 ± 0.18	**Time points**	2	0.614
								**T1**	0.86 ± 0.14	1.00 ± 0.18	0.71 ± 0.13	**FACIT-Fatigue**	1	**0.022**
								**T2**	0.99 ± 0.20	1.10 ± 0.24	0.89 ± 0.18	**Interaction**^**2**^	2	0.907
**CS-31 Total Score**	94.20 ± 5.88^a^	59.87 ± 4.86^b^	-34.337	-45.278	-23.397	37.839	** < 0.001**			72.10 ± 8.33^a^	57.24 ± 6.88^b^			
								**T0**	65.76 ± 9.17	76.13 ± 10.02	55.38 ± 9.36	**Time points**	2	0.545
								**T1**	62.05 ± 7.21	65.61 ± 9.01	58.49 ± 6.94	**FACIT-Fatigue**	1	**0.007**
								**T2**	66.21 ± 9.94	74.57 ± 11.66	57.84 ± 9.61	**Interaction**^**2**^	2	0.219
**CS-31 Menopause and Health Domain**	50.00 ± 3.08^a^	32.86 ± 2.55^b^	-17.143	-22.882	-11.405	34.284	** < 0.001**			35.17 ± 4.08^a^	29.25 ± 3.21^b^			
								**T0**	30.45 ± 4.19	33.76 ± 4.72	27.14 ± 4.35	**Time points**	2	0.771
								**T1**	31.83 ± 3.40	32.98 ± 4.44	30.69 ± 3.26	**FACIT-Fatigue**	1	**0.042**
								**T2**	34.36 ± 4.95	38.78 ± 6.08	29.93 ± 4.70	**Interaction**^**2**^	2	0.385
**CS-31 Psychological Domain**	26.25 ± 2.46^a^	10.03 ± 2.03^b^	-16.214	-20.792	-11.636	48.180	** < 0.001**			15.89 ± 3.41^a^	9.60 ± 2.76^b^			
								**T0**	17.52 ± 3.68	24.11 ± 4.05^a^	10.94 ± 3.78	**Time points**	2	0.060
								**T1**	10.60 ± 2.92	11.48 ± 3.81^b^	9.71 ± 2.79	**FACIT-Fatigue**	1	**0.008**
								**T2**	10.11 ± 3.94	12.09 ± 4.74^a,b^	8.14 ± 3.78	**Interaction**^**2**^	2	**0.004**
**CS-31 Sexuality Domain**	12.78 ± 1.58	12.53 ± 1.30	-0.250	-3.183	2.683	0.028	0.867			13.27 ± 2.29	12.29 ± 1.84			
								**T0**	11.82 ± 2.46	11.69 ± 2.73	11.95 ± 2.53	**Time points**	2	0.541
								**T1**	12.20 ± 1.95	12.45 ± 2.51	11.96 ± 1.87	**FACIT-Fatigue**	1	0.530
								**T2**	14.32 ± 2.68	15.66 ± 3.24	12.98 ± 2.56	**Interaction**^**2**^	2	0.494
**CS-31 Couple Relations Domain**	5.08 ± 1.46	4.49 ± 1.20	-0.591	-3.302	2.120	0.182	0.669			9.27 ± 1.79^a^	6.17 ± 1.48^b^			
								**T0**	6.31 ± 1.96	7.52 ± 2.13	5.10 ± 2.01	**Time points**	2	0.355
								**T1**	8.47 ± 1.56	10.34 ± 2.06	6.59 ± 1.50	**FACIT-Fatigue**	1	**0.008**
								**T2**	8.38 ± 2.05	9.95 ± 2.40	6.81 ± 1.99	**Interaction**^**2**^	2	0.766
**CS-31 Vasomotor Subdomain**	11.02 ± 1.20^a^	8.74 ± 0.99^b^	-2.279	-4.509	-0.050	4.015	**0.045**			7.28 ± 1.68^a^	6.80 ± 1.33^b^			
								**T0**	6.59 ± 1.78	6.56 ± 1.99	6.63 ± 1.85	**Time points**	2	0.922
								**T1**	7.15 ± 1.42	7.11 ± 1.92	7.19 ± 1.36	**FACIT-Fatigue**	1	0.684
								**T2**	7.37 ± 1.90	8.16 ± 2.36	6.58 ± 1.81	**Interaction**^**2**^	2	0.647
**CS-31 Health Subdomain**	16.75 ± 1.17^a^	10.42 ± 0.97^b^	-6.325	-8.513	-4.138	32.115	** < 0.001**			11.30 ± 1.92^a^	7.91 ± 1.47^b^			
								**T0**	9.87 ± 1.98	12.36 ± 2.26	7.40 ± 2.06	**Time points**	2	0.502
								**T1**	8.83 ± 1.58	9.07 ± 2.12	8.59 ± 1.51	**FACIT-Fatigue**	1	**0.019**
								**T2**	10.12 ± 2.20	12.48 ± 2.84	7.76 ± 2.06	**Interaction**^**2**^	2	0.097
**CS-31 Aging Subdomain**	22.24 ± 1.74^a^	13.70 ± 1.44^b^	-8.539	-11.777	-5.301	26.721	** < 0.001**			17.19 ± 2.25^a^	14.09 ± 1.85^b^			
								**T0**	14.29 ± 2.47	15.40 ± 2.71	13.19 ± 2.52	**Time points**	2	0.727
								**T1**	15.66 ± 1.94	17.26 ± 2.39	14.07 ± 1.87	**FACIT-Fatigue**	1	**0.036**
								**T2**	16.97 ± 2.72	18.92 ± 3.22	15.02 ± 2.62	**Interaction**^**2**^	2	0.765
**CS-31 Aching in muscles and/or joints**	4.19 ± 0.35^a^	3.41 ± 0.31^b^	-0.779	-1.394	-0.165	6.176	**0.013**			3.79 ± 0.49	3.29 ± 0.39			
								**T0**	3.33 ± 0.51	3.25 ± 0.56	3.41 ± 0.52	**Time points**	2	0.502
								**T1**	3.40 ± 0.44	3.73 ± 0.60	3.07 ± 0.41	**FACIT-Fatigue**	1	0.110
								**T2**	3.89 ± 0.56	4.40 ± 0.67	3.39 ± 0.53	**Interaction**^**2**^	2	0.057

^*^Generalized linear models (GLzM); **General Mixed Model (GMM); Data adjusted for age, education level, income, usage time of aromatase inhibitors and body mass index. FACIT-Fatigue, Functional Assessment of Chronic Illness Therapy—Fatigue Scale, with cut-off < 34 indicating cancer-related fatigue; Time point: T0, Baseline; T1, Intermediate period, corresponding to 12 months after T0; and T2, Final follow-up period, corresponding to 24 months after T0; PRO Instrument, Patient-Reported Outcome Instrument; HADS-A: Hospital Anxiety and Depression Scale, subscale anxiety; HADS-D: Hospital Anxiety and Depression Scale, subscale depression; HAQ, Health Assessment Questionnaire; CS-31, 31-item Cervantes Scale; SD, Standard deviation; Df, degrees of freedom. Significant Model Effects Test are in bold. Sidak test: Different superscript letters represent statistical significance when comparing pairs, p value < 0.05. ^1^Longitudinal phase: PRO Instruments are shown as mean ± SD; ^2^Interaction between FACIT-Fatigue and time points of study

Longitudinally, the CRF had negative effect on Couple Relations domain (*p* = 0.008), and the significances were maintained for anxiety (*p* = 0.028), depression (*p* = 0.027), functional disability (*p* = 0.022), HRQL (*p* = 0.007), Menopause and Health (*p* = 0.042), Psychological (*p* = 0.008), Health (p = 0.019) and Aging (*p* = 0.036) (Table 5).

The time of study had effect on anxiety (*p* = 0.035), with the significance indicating worse score in T0 compared T1 (Table 5). In addition, the interaction between FACIT-Fatigue and time points of study had effect on Psychological domain (*p* = 0.004), with significance in the CRF subgroup, indicating a worse score in this domain in T0 compared T1 (Table 5).

## Discussion

Our results showed that slightly more than one-third of the BC survivors had CRF and low serum 25(OH)D levels. The women with CRF had been using AI for a shorter time when compared to the subgroup without CRF. The dietary intake of vitamin D did not differ significantly between the 25(OH) D levels subgroups, however none of the women reached the EAR of this nutrient. There were negative associations between the FACIT-Fatigue score and body adiposity (BMI, WC, WHtR, body fat and conicity index). The greater percentage of women was classified as “better”, i.e. with improvement in the FACIT-Fatigue score throughout the study. No significance was found regarding the direct and reverse causality in the association between 25(OH)D concentration and CRF. As for PRO Instruments, lower 25(OH)D level had a negative effect on the scores of anxiety, Menopause and Health domain, and Vasomotor subdomain. In addition, those women with clinically relevant CRF presented more anxiety, depression, functional disability, muscle/joint aches, worse HRQL and worse score in the following domains and subdomains of the CS-31 – Menopause and health, Psychological, Vasomotor, Health, Aging and Couple Relations. These results confirm our initial hypothesis and show the clinical relevance of both 25(OH)D and CRF, highlighting the latter.

Many factors may influence the bioavailability of vitamin D, such as changes in the physiochemical state of this vitamin, complexity of the food matrix, interaction of the vitamin D with other fat-soluble compounds and individual factors [58]. In our study, the 25(OH)D level was positively associated with the MUFA/PUFA ratio and negatively associated with omega-6 intake, which is similar to results found in a study that investigated the change in 25(OH)D level after vitamin D supplementation in healthy older adults [59]. Dawson-Hughes and colleagues identified that the presence of fat in meals increased the absorption of vitamin D from a supplement, but they did not find influence of MUFA/PUFA ratio [60]. The mechanisms proposed by Hollander and colleagues suggest that long-chain fatty acids (oleic and linoleic acids) increase micelle size, impairing the passive diffusion of vitamin D through enterocytes, unlike short (butyric acid) and medium-chain fatty acids (octanoic acid), which are water soluble and do not require micellar formation for their absorption [61]. Although these authors found that the greater degree of unsaturation of fatty acids slowed the rate of vitamin D absorption in the gut [61], more evidence is needed to confirm and explain the mechanism by the MUFA/PUFA ratio would influence the 25(OH)D level.

The dietary intake of vitamin D is commonly low among BC women (mean 4.7 µg/day [62]), as we identified in our study, in which the mean intake of this nutrient was 3.7 ± 1.7 µg/day. A meta-analysis including 10 prospective cohort studies and totalizing 22,341 BC incident cases identified that the lowest categories of vitamin D intake presented a mean below 148 IU/day (3.7 µg/day) [63]. The Recommended Dietary Allowances (RDA) for females until 70 years of age is 15 µg/day and older is 20 µg/day, reference values assuming minimal sun exposure [57]. Food is not the unique source of 25(OH)D, being the serum concentration strongly influenced by direct exposure of the skin to sunlight [64]. However, some factors may influence the sun induced synthesis, among which the season, which influenced the 25(OH)D levels in the present study; time of day; latitude; altitude; air pollution; skin pigmentation; sunscreen; aging; sunlight passing through glass and plastic [64]. Moreover, some individuals are exposed to very limited amounts of solar ultraviolet radiation, making them dependent on an adequate oral intake of this vitamin, in order to favor optimal 25(OH)D levels [65–67]. Amrein and colleagues [68] defend the importance of vitamin D supplementation in certain risk groups and the vitamin D food fortification as a worldwide public health strategy to avoid severe vitamin D deficiency.

Although there is no consensus regarding the optimal 25(OH)D level in the body, the SBEM and SBPC in a position statement about the reference values, identify the potential benefits of maintaining 25(OH)D levels above 75 nmol/L in specific conditions, reference value in accord with the Endocrine Society [69]. Among the specific conditions, we highlight the elderly, individuals with cancer and using drugs with the potential to affect the vitamin D metabolism, characteristics present in our sample [42]. Moreover, the use of AIs (letrozole and exemestane) could also increase the requirements for this vitamin, considering that they are metabolized in the liver by the CYP3A4 system [70] and the vitamin D induce the expression of these genes [71]. In our study, the median 25(OH)D level was 84 nmol/L (range 18.5 – 137.3 nmol/L) and 39.3% (*n* = 35) presented 25(OH)D levels below 75 nmol/L, similar result to the study of Friedman and colleagues, in which the median was 87.5 nmol/L (range 17 – 232.9 nmol/L) and 35% (*n* = 136) of postmenopausal BC survivors presented level < 75nmoL/L [72]. According to the US Institute of Medicine, 25(OH)D levels equal to or above 50 nmol/L is sufficient for practically all persons for proper functioning of the calcium-phosphate metabolism and to maintain bone density and there would be no increased benefit in 25(OH)D levels above 75 nmol/L [57]. However, evidence suggest potential additional benefits for levels above 75 nmol/L as a reduction in the risk of fractures [42] and falls, better tooth attachment, improved depression and wellbeing [8], reduction of the risk of autoimmune diseases, type 2 diabetes, cardiovascular disease, infectious diseases [69] and neoplastic diseases [8, 69]. High 25(OH)D levels were significantly associated with lower BC mortality (> 72.75 vs < 52.5 nmol/L, pooled RR = 0.58, 95% CI: 0.40–0.85), overall mortality (> 68.75 vs < 51.75 nmol/L, pooled RR = 0.61, 95% CI: 0.48–0.79) and BC recurrence (> 67.25 vs < 36.75 nmol/L, pooled RR = 0.61, 95% CI: 0.47–0.80) [63]. Calcitriol (1,25-dihydroxyvitamin D3), the active metabolite of vitamin D, present anticancer actions as cell cycle arrest, stimulation of apoptosis and inhibition of invasion, metastasis and angiogenesis, inhibiting the growth of malignant cells including BC cells [73].

Cancer treatment-induced bone loss is a common side effect in BC women [74]. The prevalence of BC women undergoing AI with adequate bone health and vitamin D status is very low, only 5.6%, which justifies the monitoring of these parameters during and even after treatment [74]. The depletion of estrogen resulting from the AI treatment may cause an accelerated decrease in bone mineral density (BMD), which is the primary cause of the increased fracture risk [75]. Patients with a high risk of fractures [75] and, or patients receiving antiresorptive drugs for cancer treatment induced bone loss, may benefit from pharmacological intervention that contribute to the preservation of bone health [74], such as vitamin D supplementation. However, although vitamin D supplementation is one of the most frequent therapies indicated to women with postmenopausal osteoporosis presenting slow BMD loss, this therapy may not be effective for the prevention of accelerated BMD loss derived from AI use [75].

Endocrine therapy is recommended for a minimum of 5 years. The option of extended endocrine therapy (EET), either with 10 years of tamoxifen or 5 years of an AI after 4.5 to 6 years of tamoxifen, has been increasingly recommended. The use of 10 years of AI has been disfavored because of the adverse event profile [76]. Although the EET with AI is associated with increased risk of bone-related toxic effects, cardiovascular events, hot flashes, arthralgia and myalgia, the EET did not increase the risk of other adverse effects, such as fatigue [77]. In our study, at baseline, the women with longer median time using AI did not present CRF and, considering the longitudinal phase, most women were classified as “better”, i.e. with improvement in FACIT-Fatigue score throughout the study. It is noteworthy that at baseline, only three women (3.4%) were on EET, i.e., in use AI beyond 5 years of therapy, already in T2, 14 women (36.8%) were on EET.

In the present study, indicators of greater body adiposity were negatively associated with the FACIT-Fatigue score, i.e., with higher CRF. Adipose tissue is a metabolically very active endocrine tissue that influences the inflammatory process [78], which was the mechanism used to explain greater CRF in obese BC patients [79]. BMI, sedentary lifestyle and nutritional deficiencies are some of risk factors associated with chronic inflammation, which have potential to generate a pre-treatment inflammatory state and even pre-treatment fatigue, identified as the principal predictor of CRF [80]. However, various mechanisms are involved in its pathophysiology, such as changes in adenosine triphosphate and muscle metabolism, neurotransmitter dysregulation, hypothalamic–pituitary–adrenal axis disruption, and neural-immune signaling triggered by inflammation [80, 81], and CRF may occurs even among patients without risk factors [80], remaining in progress investigations regarding its etiology.

The causal effect of body adiposity on the CRF cannot be confirmed in the present study, yet the result is alarming considering that obesity and abdominal visceral adipose tissue accumulation are associated with metabolic consequences and risk of cardiovascular disease (CVD), the latter being the leading cause of death in postmenopausal women [82] and also in BC survivors [83]. Furthermore, antineoplastic treatment, including endocrine therapy, may favor cardiotoxicity [84]. Mazzutti and colleagues [30] identified in the same sample of the present study, that women in AI use had a significant number of risk factors for CVD. Considering only the risk factor “body adiposity”, it is reasonable to infer that women with CRF would be at increased risk of metabolic syndrome and other cardiovascular disorders when compared to those without CRF, which deserves further consideration.

The CRF affects 50% to 90% of cancer patients regardless of age, sex or diagnosis [85], persisting after the end of treatment [86, 87] and presenting emotional, physical, cognitive, functional consequences, in addition to causing uncertainty and impact the sense-of-self [88]. In the present study, CRF was associated with practically all health outcomes investigated, being relevant result for clinical practice and reinforces the need for more studies aimed at the development of effective interventions to control this adverse effect whit high potential for better the HRQL and associated aspects.

Testing and correction of 25(OH)D levels are commonly studied in cases of fatigue [7, 89–93], but more studies are needed to prove the effectiveness of vitamin D supplementation in reducing or preventing of the CRF. The VICTORIA study aims to confirm this association through a randomized controlled trial [94]. A recent genetic analysis between low 25(OH)D levels and fatigue showed little evidence of a causal effect, suggesting an unlikely protective effect of 25(OH)D on fatigue, but considering a lifelong exposure to a low serum concentration, unlike observational or intervention studies which investigate the association between short-term changes in 25(OH)D concentration and fatigue [95]. Havdahl, Paternoster and Smith [95] believe that there is a reverse causality between fatigue and 25(OH)D levels, and that this last could be just one marker or consequence of fatigue, considering that fatigued individuals tend to sedentary lifestyle and longer stay indoors without exposure to the sun. In addition, these authors emphasize that fatigue and 25(OH)D deficiency have risk factors in common, which can favor misinterpretations without proper adjustments [95]. In our prospective study, there was no significant causality between 25(OH)D levels and CRF, but the effect of both on important aspects of HRQL in BC survivors is notable, therefore not disregarding the favorable effects of a possible supplementation of vitamin D in this population.

We must consider some limitations, as a small sample size and sample loss during follow-up. Furthermore, the assessment of food consumption from 24HR is subject to memory bias, although the interviews were conducted by nutritionists to minimize this risk. Pharmaceutical history might influence functional outcomes and improve the sample characterization, but unfortunately these data were not collected completely. In addition, the PRO Instruments used in this study are self-reported questionnaires, however all participants replied by interview, which may have inhibited responses to certain items. This standardization was necessary considering there were illiterate women in our sample, being the interviews conducted by properly trained researchers. Due to the intrinsic limitations of a monocentric design, these results cannot be generalized for the general population, although they are relevant and may be useful in the elaboration of hypotheses for future larger studies and multicentric investigations. A strength of this study is the assessment of CRF, 25(OH)D, anxiety, depression, functional disability, muscle/joint aches, HRQL and related aspects at three time points, with a 2-year follow-up. Furthermore, to the best of our knowledge, this is the first study to assess both the association and the effect of vitamin D and CRF on several aspects related to the health of BC survivors using AI.

Maintaining quality of life and fatigue were the principal global issue and the specific symptom, respectively, identified by BC patients [96]. However, the fatigue is underreported as cancer patients frequently associate this complication with disease progression or treatment ineffectiveness rather than as an adverse effect of treatment, and the fear of progression inhibits reporting [17]. In addition, some physicians have insufficient knowledge about CRF, available pharmacologic and nonpharmacologic interventions, as well as their serious consequences on HRQL [85]. This reinforces the need for wide dissemination in academic and scientific environment, counseling both patient and family, and monitoring this adverse effect on oncology medical routine through appropriate instruments, in order to contribute to improving HRQL and health outcomes [17].

## Conclusions

The clinical relevance of 25(OH)D and CRF is highlighted, especially of the CRF, considering the consistent impact on several adverse effects often reported by women in AI use, such as anxiety, depression, functional disability, muscle/joint aches, HRQL, couple relations, psychological symptoms, and related to menopause and health. In addition, it is important to note the negative effect of body adiposity on CRF. Strategies that comprise emotional support, physical exercise, and nutritional guidance need to be included in routine care of BC survivors during adjuvant endocrine therapy. More studies aimed at the development of feasible and effective interventions are awaited.

## Supplementary Information

**Additional file 1:** **Supplementary Table 1.** Demographic and clinical characteristics of the breast cancer survivors during endocrine therapy, considering FACIT-Fatigue subgroups.

**Additional file 2:** **Supplementary Table 2.** Direct and reverse association between 25(OH)D and cancer-related fatigue.

## Data Availability

The datasets used and/or analyzed during the current study are available from the corresponding author on reasonable request.

## Abbreviations

24HR: 24-Hour dietary recall
25(OH)D: 25-Hydroxyvitamin D
AI: Aromatase Inhibitors
AIC: Akaike Information Criterion
BC: Breast cancer
BF: Body fat
BIA: Bioelectrical impedance analysis
BMI: Body mass index
CRF: Cancer-related fatigue
CS: Cervantes Scale
CS-31: 31-Item Cervantes Scale
CVD: Cardiovascular disease
EAR: Estimated Average Requirements
EET: Extended endocrine therapy
FACIT-F: Functional Assessment of Chronic Illness Therapy-fatigue
FACT-G: Functional Assessment of Cancer Therapy-General
GLzM: Generalized Linear Model
GMM: Generalized Mixed Model
HADS: Hospital Anxiety and Depression Scale
HADS-A: Hospital Anxiety and Depression Scale, subscale anxiety
HADS-D: Hospital Anxiety and Depression Scale, subscale depression
HAQ: Health Assessment Questionnaire
HR +: Receptor-positive
HRQL: Health-Related Quality of Life
MCID: Minimum Clinically Important Difference
MUFA: Monounsaturated fatty acids
NDSR: Nutrition Data System for Research
PRO: Patient-reported outcome
PUFA: Polyunsaturated fatty acids
RDA: Recommended Dietary Allowances
SBEM: Brazilian Society of Endocrinology and Metabolism
SBPC: Brazilian Society of Clinical Pathology/Laboratory Medicine
T0: Baseline
T1: Intermediate follow-up period
T2: Final follow-up period
WC: Waist circumference
WHR: Waist-to-hip ratio
WHtR: Waist-to-height ratio

## References

[1] Ferlay J, Ervik M, Lam F, Colombet M, Mery L, Piñeros M, Znaor A, Soerjomataram I, Bray F. Global Cancer Observatory: Cancer Today. Lyon, France: International Agency for Research on Cancer. 2020. https://gco.iarc.fr/today. Accessed 17 Sep 2021.

[2] Smith I, Dowsett M. Aromatase inhibitors in breast cancer. N Engl J Med. 2003. 10.1056/NEJMra023246 12802030

[3] Condorelli R, Vaz-Luis I (2018). Managing side effects in adjuvant endocrine therapy for breast cancer. Expert Rev Anticancer Ther.

[4] Dahir M, Travers-Gustafson D (2014). Breast cancer, aromatase inhibitor therapy, and sexual functioning: a pilot study of the effects of vaginal testosterone therapy. Sex Med.

[5] Hines SL, Jorn HKS, Thompson KM, Larson JM (2010). Breast cancer survivors and vitamin D: A review. Nutrition.

[6] Kwan ML, Lo JC, Tang L, Laurent CA, Roh JM, Chandra M (2014). Bone Health History in Breast Cancer Patients on Aromatase Inhibitors. PLoS ONE.

[7] Khan QJ, Reddy PS, Kimler BF, Sharma P, Baxa SE, O’Dea AP (2010). Effect of vitamin D supplementation on serum 25-hydroxy vitamin D levels, joint pain, and fatigue in women starting adjuvant letrozole treatment for breast cancer. Breast Cancer Res Treat.

[8] Vieth R (2011). Why the minimum desirable serum 25-hydroxyvitamin D level should be 75 nmol/L (30 ng/ml). Best Pract Res Clin Endocrinol Metab.

[9] Polak MA, Houghton LA, Reeder AI, Harper MJ, Conner TS (2014). Serum 25-Hydroxyvitamin D Concentrations and Depressive Symptoms among Young Adult Men and Women. Nutrients.

[10] Holick MF, Vitamin D (2007). Deficiency. N Engl J Med.

[11] ED Michos ML Melamed 2008 Vitamin D and cardiovascular disease risk: Curr Opin Clin Nutr Metab Care 11 7 12 10.1097/MCO.0b013e3282f2f4dd 18090651

[12] Buchanan JR, Santen R, Cauffman S, Cavaliere A, Greer RB (1986). The effect of endogenous estrogen fluctuation on metabolism of 25-hydroxyvitamin D. Calcif Tissue Int.

[13] Ellis SD, Kelly ST, Shurlock JH, Hepburn ALN (2018). The role of vitamin D testing and replacement in fibromyalgia: a systematic literature review. BMC Rheumatol.

[14] McCann JC, Ames BN (2008). Is there convincing biological or behavioral evidence linking vitamin D deficiency to brain dysfunction?. FASEB J.

[15] Garcion E, Wion-Barbot N, Montero-Menei CN, Berger F, Wion D (2002). New clues about vitamin D functions in the nervous system. Trends Endocrinol Metab.

[16] Henry NL, Azzouz F, Desta Z, Li L, Nguyen AT, Lemler S (2012). Predictors of Aromatase Inhibitor Discontinuation as a Result of Treatment-Emergent Symptoms in Early-Stage Breast Cancer. J Clin Oncol.

[17] National Comprehensive Cancer Network. NCCN guidelines, version 2, 2022. Cancer-related fatigue. Available at: https://www.nccn.org/guidelines/guidelines-detail?category=3&id=1424. Accessed: May 2022.

[18] Curt GA, Breitbart W, Cella D, Groopman JE, Horning SJ, Itri LM (2000). Impact of cancer-related fatigue on the lives of patients: new findings from the Fatigue Coalition. Oncologist.

[19] Ruiz-Casado A, Álvarez-Bustos A, de Pedro CG, Méndez-Otero M, Romero-Elías M (2021). Cancer-related Fatigue in Breast Cancer Survivors: A Review. Clin Breast Cancer.

[20] Maass SWMC, Brandenbarg D, Boerman LM, Verhaak PFM, de Bock GH, Berendsen AJ. Fatigue among Long-Term Breast Cancer Survivors: A Controlled Cross-Sectional Study. Cancers. 2021;13. 10.3390/cancers13061301 PMC8001130 33803966

[21] Invernizzi M, de Sire A, Lippi L, Venetis K, Sajjadi E, Gimigliano F (2020). Impact of Rehabilitation on Breast Cancer Related Fatigue: A Pilot Study. Front Oncol.

[22] Jang A, Brown C, Lamoury G, Morgia M, Boyle F, Marr I (2020). The Effects of Acupuncture on Cancer-Related Fatigue: Updated Systematic Review and Meta-Analysis. Integr Cancer Ther.

[23] O’Neill M, Samaroo D, Lopez C, Tomlinson G, Santa Mina D, Sabiston C (2020). The Effect of Yoga Interventions on Cancer-Related Fatigue and Quality of Life for Women with Breast Cancer: A Systematic Review and Meta-Analysis of Randomized Controlled Trials. Integr Cancer Ther.

[24] Haller H, Winkler MM, Klose P, Dobos G, Kümmel S, Cramer H (2017). Mindfulness-based interventions for women with breast cancer: an updated systematic review and meta-analysis. Acta Oncol.

[25] Corbett TK, Groarke A, Devane D, Carr E, Walsh JC, McGuire BE (2019). The effectiveness of psychological interventions for fatigue in cancer survivors: systematic review of randomised controlled trials. Syst Rev.

[26] Poort H, Peters MEWJ, van der Graaf WTA, Nieuwkerk PT, van de Wouw AJ, der Sanden MWGN (2020). Cognitive behavioral therapy or graded exercise therapy compared with usual care for severe fatigue in patients with advanced cancer during treatment: a randomized controlled trial. Ann Oncol.

[27] Bennett S, Pigott A, Beller EM, Haines T, Meredith P, Delaney C. Educational interventions for the management of cancer-related fatigue in adults. Cochrane Database Syst Rev. 2016;11:CD008144. 10.1002/14651858.CD008144.pub2 PMC6464148 27883365

[28] Klasson C, Helde Frankling M, Warnqvist A, Sandberg C, Nordström M, Lundh-Hagelin C (2022). Sex Differences in the Effect of Vitamin D on Fatigue in Palliative Cancer Care—A Post Hoc Analysis of the Randomized, Controlled Trial ‘Palliative-D’. Cancers.

[29] Joly F, Lange M, Dos Santos M, Vaz-Luis I, Di Meglio A (2019). Long-Term Fatigue and Cognitive Disorders in Breast Cancer Survivors. Cancers.

[30] Mazzutti FS, Custódio IDD, Lima MTM, Carvalho KP de, Pereira TSS, Molina M del CB, et al. Breast Cancer Survivors Undergoing Endocrine Therapy Have a Worrying Risk Factor Profile for Cardiovascular Diseases. Nutrients. 2021;13:1114. 10.3390/nu13041114 PMC8067236 33805280

[31] Lohman TG, Roche AF, Martorell R (1988). Anthropometric Standardization Reference Manual.

[32] World Health Organization. Obesity: preventing and managing the global epidemic. No. 894. 2000. 11234459

[33] Ashwell M, Hsieh SD (2005). Six reasons why the waist-to-height ratio is a rapid and effective global indicator for health risks of obesity and how its use could simplify the international public health message on obesity. Int J Food Sci Nutr.

[34] Rato Q (2017). Índice de conicidade: uma medida antropométrica a avaliar. Rev Port Cardiol.

[35] Lipschitz DA (1994). Screening for nutritional status in the elderly. Prim Care.

[36] Associação Brasileira de Nutrologia, Sociedade Brasileira de Nutrição Parenteral e Enteral. Utilização da Bioimpedância para Avaliação da Massa Corpórea. 2009;13.

[37] Kyle UG, Bosaeus I, De Lorenzo AD, Deurenberg P, Elia M, Gómez JM (2004). Bioelectrical impedance analysis—part I: review of principles and methods. Clin Nutr.

[38] Kyle UG, Genton L, Karsegard L, Slosman DO, Pichard C (2001). Single prediction equation for bioelectrical impedance analysis in adults aged 20–94 years. Nutr (Burbank, Los Angel Cty, Calif).

[39] Brasil. Ministério da Saúde. Secretaria de Vigilância em Saúde. Departamento de Análise em Saúde e Vigilância de Doenças, Não Transmissíveis. Vigitel Brasil 2019: Vigilância de Fatores de Risco e Proteção para Doenças Crônicas por Inquérito Telefônico: Estimativas sobre Frequência e Distribuição Sociodemográfica de Fatores de Risco e Proteção para Doenças Crônicas nas Capitais dos 26 Estados Brasileiros e no Distrito Federal em 2019. http://bvsms.saude.gov.br/bvs/publicacoes/vigitel_brasil_2019_vigilancia_fatores_risco.pdf. Accessed 27 Jul 2021.

[40] Nusser SM, Carriquiry AL, Dodd KW, Fuller WA (1996). A Semiparametric Transformation Approach to Estimating Usual Daily Intake Distributions. J Am Stat Assoc.

[41] Willett WC, Howe GR, Kushi LH. Adjustment for total energy intake in epidemiologic studies. Am J Clin Nutr. 1997;65 4 Suppl:1220S-1228S; discussion 1229S-1231S. 10.1093/ajcn/65.4.1220S 9094926

[42] Moreira CA, Ferreira CE dos S, Madeira M, Silva BCC, Maeda SS, Batista MC, et al. Reference values of 25-hydroxyvitamin D revisited: a position statement from the Brazilian Society of Endocrinology and Metabolism (SBEM) and the Brazilian Society of Clinical Pathology/Laboratory Medicine (SBPC). Arch Endocrinol Metab. 2020;64:462–78. 10.20945/2359-3997000000258 PMC10522078 32813765

[43] Palacios , Ferrer-Barriendos  J, José Parrilla  J, Castelo-Branco  C, Manubens  M , Alberich  X (2004). Calidad de vida relacionada con la salud en la mujer española durante la perimenopausia y posmenopausia. Desarrollo y validación de la Escala Cervantes. Med Clínica.

[44] Lima JEM, Palacios S, Wender MCO (2012). Quality of Life in Menopausal Women: A Brazilian Portuguese Version of the Cervantes Scale. Sci World J.

[45] Ishikawa NM, Thuler LCS, Giglio AG, Baldotto CS da R, de Andrade CJC, Derchain SFM. Validation of the Portuguese version of Functional Assessment of Cancer Therapy-Fatigue (FACT-F) in Brazilian cancer patients. Support Care Cancer. 2010;18:481–90. 10.1007/s00520-009-0697-0 19629540

[46] Yellen SB, Cella D, Webster K, Blendowski C, Kaplan E (1997). Measuring fatigue and other anemia-related symptoms with the Functional Assessment of Cancer Therapy (FACT) measurement system. J Pain Symptom Manage.

[47] Van Belle S, Paridaens R, Evers G, Kerger J, Bron D, Foubert J (2005). Comparison of proposed diagnostic criteria with FACT-F and VAS for cancer-related fatigue: proposal for use as a screening tool. Support Care Cancer.

[48] Zigmond AS, Snaith RP (1983). The Hospital Anxiety and Depression Scale. Acta Psychiatr Scand.

[49] Botega NJ, Bio MR, Zomignani MA, Garcia C, Pereira WAB (1995). Transtornos do humor em enfermaria de clínica médica e validação de escala de medida (HAD) de ansiedade e depressão. Rev Saúde Pública.

[50] Fries JF, Spitz P, Kraines RG, Holman HR (1980). Measurement of patient outcome in arthritis. Arthritis Rheum.

[51] Bruce B, Fries JF (2005). The Health Assessment Questionnaire (HAQ). Clin Exp Rheumatol.

[52] Bruce B, Fries JF. The Stanford Health Assessment Questionnaire: Dimensions and Practical Applications. Health Qual Life Outcomes. 2003;6. 10.1186/1477-7525-1-20 PMC165587 12831398

[53] Ferraz MB, Oliveira LM, Araujo PM, Atra E, Tugwell P (1990). Crosscultural reliability of the physical ability dimension of the health assessment questionnaire. J Rheumatol.

[54] Faul F, Erdfelder E, Lang A-G, Buchner A (2007). G*Power 3: A flexible statistical power analysis program for the social, behavioral, and biomedical sciences. Behav Res Methods.

[55] Terwee CB, Bot SDM, de Boer MR, van der Windt DAWM, Knol DL, Dekker J (2007). Quality criteria were proposed for measurement properties of health status questionnaires. J Clin Epidemiol.

[56] Fayers P, Machin D. Quality of Life: The Assessment, Analysis and Interpretation of Patients-reported Outcomes. 2nd edition. Chinchester, UK: John Willey & Sons; 2007.

[57] Institute of Medicine (US) Committee to Review Dietary Reference Intakes for Vitamin D and Calcium. Dietary Reference Intakes for Calcium and Vitamin D. Washington (DC): National Academies Press (US); 2011. http://www.ncbi.nlm.nih.gov/books/NBK56070/. Accessed 8 Sep 2021. 21796828

[58] Maurya VK, Aggarwal M (2017). Factors influencing the absorption of vitamin D in GIT: an overview. J Food Sci Technol.

[59] Niramitmahapanya S, Harris SS, Dawson-Hughes B (2011). Type of Dietary Fat Is Associated with the 25-Hydroxyvitamin D3 Increment in Response to Vitamin D Supplementation. J Clin Endocrinol Metab.

[60] Dawson-Hughes B, Harris SS, Lichtenstein AH, Dolnikowski G, Palermo NJ, Rasmussen H (2015). Dietary fat increases vitamin D-3 absorption. J Acad Nutr Diet.

[61] Hollander D, Muralidhara KS, Zimmerman A (1978). Vitamin D-3 intestinal absorption in vivo: influence of fatty acids, bile salts, and perfusate pH on absorption. Gut.

[62] Saquib J, Rock CL, Natarajan L, Saquib N, Newman VA, Patterson RE (2011). Dietary Intake, Supplement Use, and Survival Among Women Diagnosed with Early Stage Breast Cancer. Nutr Cancer.

[63] Kim Y, Je Y (2014). Vitamin D intake, blood 25(OH)D levels, and breast cancer risk or mortality: a meta-analysis. Br J Cancer.

[64] Wacker M, Holick MF (2013). Sunlight and Vitamin D Dermatoendocrinol.

[65] Glerup H, Mikkelsen K, Poulsen L, Hass E, Overbeck S, Thomsen J (2000). Commonly recommended daily intake of vitamin D is not sufficient if sunlight exposure is limited. J Intern Med.

[66] Holick MF (1994). McCollum Award Lecture, 1994: Vitamin D—new horizons for the 21st century. Am J Clin Nutr.

[67] Webb AR, Pilbeam C, Hanafin N, Holick MF (1990). An evaluation of the relative contributions of exposure to sunlight and of diet to the circulating concentrations of 25-hydroxyvitamin D in an elderly nursing home population in Boston. Am J Clin Nutr.

[68] Amrein  K, Scherkl  M, Hoffmann  M, Neuwersch-Sommeregger  S, Köstenberger  M, Tmava Berisha  A (2020). Vitamin D deficiency 2.0: an update on the current status worldwide. Eur J Clin Nutr.

[69] Holick MF, Binkley NC, Bischoff-Ferrari HA, Gordon CM, Hanley DA, Heaney RP (2011). Evaluation, Treatment, and Prevention of Vitamin D Deficiency: an Endocrine Society Clinical Practice Guideline. J Clin Endocrinol Metab.

[70] Arora A, Potter JF (2004). Aromatase Inhibitors: Current Indications and Future Prospects for Treatment of Postmenopausal Breast Cancer: AROMATASE INHIBITORS IN LATE-LIFE BREAST CANCER. J Am Geriatr Soc.

[71] Drocourt L, Ourlin J-C, Pascussi J-M, Maurel P, Vilarem M-J (2002). Expression of CYP3A4, CYP2B6, andCYP2C9 Is Regulated by the Vitamin D Receptor Pathway in Primary Human Hepatocytes *. J Biol Chem.

[72] Friedman CF, DeMichele A, Su HI, Feng R, Kapoor S, Desai K (2012). Vitamin D Deficiency in Postmenopausal Breast Cancer Survivors. J Womens Health.

[73] Krishnan AV, Swami S, Feldman D (2012). The Potential Therapeutic Benefits of Vitamin D in the Treatment of Estrogen Receptor Positive Breast Cancer. Steroids.

[74] de Sire A, Gallelli L, Marotta N, Lippi L, Fusco N, Calafiore D (2022). Vitamin D Deficiency in Women with Breast Cancer: A Correlation with Osteoporosis? A Machine Learning Approach with Multiple Factor Analysis. Nutrients.

[75] Body J-J (2011). Increased fracture rate in women with breast cancer: a review of the hidden risk. BMC Cancer.

[76] Gradishar WJ, Moran MS, Abraham J, Aft R, Agnese D, Allison KH et al. Breast Cancer, Version 5.2021, NCCN Clinical Practice Guidelines in Oncology (NCCN Guidelines^®^). 2021. https://www.nccn.org/professionals/physician_gls/pdf/breast.pdf. Accessed 31 Jul 2021.

[77] Zhao F, Ren D, Shen G, Ahmad R, Dong L, Du F (2020). Toxicity of extended adjuvant endocrine with aromatase inhibitors in patients with postmenopausal breast cancer: A Systemtic review and Meta-analysis. Crit Rev Oncol Hematol.

[78] Izaola O, de Luis D, Sajoux I, Domingo JC (2015). Vidal M [Inflammation and obesity (lipoinflammation)]. Nutr Hosp.

[79] Inglis JE, Janelsins MC, Culakova E, Mustian KM, Lin P-J, Kleckner IR (2020). Longitudinal assessment of the impact of higher body mass index on cancer-related fatigue in patients with breast cancer receiving chemotherapy. Support Care Cancer Off J Multinatl Assoc Support Care Cancer.

[80] Bower JE (2014). Cancer-related fatigue: Mechanisms, risk factors, and treatments. Nat Rev Clin Oncol.

[81] O’Higgins CM, Brady B, O’Connor B, Walsh D, Reilly RB (2018). The pathophysiology of cancer-related fatigue: current controversies. Support Care Cancer.

[82] Kapoor E, Collazo-Clavell ML, Faubion SS (2017). Weight Gain in Women at Midlife: A Concise Review of the Pathophysiology and Strategies for Management. Mayo Clin Proc.

[83] Bradshaw PT, Stevens J, Khankari N, Teitelbaum SL, Neugut AI, Gammon MD (2016). Cardiovascular Disease Mortality Among Breast Cancer Survivors. Epidemiol Camb Mass.

[84] Sharma AV, Reddin G, Forrestal B, Barac A (2019). Cardiovascular Disease Risk in Survivors of Breast Cancer. Curr Treat Options Cardiovasc Med.

[85] Campos MPO, Hassan BJ, Riechelmann R, Del Giglio A (2011). Cancer-related fatigue: a practical review. Ann Oncol Off J Eur Soc Med Oncol.

[86] Bower JE, Ganz PA, Desmond KA, Bernaards C, Rowland JH, Meyerowitz BE (2006). Fatigue in long-term breast carcinoma survivors. Cancer.

[87] Coughlin SS, Ayyala DN, Cortes JE (2021). Problems in living among breast cancer survivors. Curr Cancer Rep.

[88] Dolgoy ND, Krishnasamy M, McNeely ML (2019). Uncertainty and sense-of-self as targets for intervention for cancer-related fatigue. Eur J Cancer Care (Engl).

[89] Beckmann Y, Türe S, Duman SU (2020). Vitamin D deficiency and its association with fatigue and quality of life in multiple sclerosis patients. EPMA J.

[90] Kerley CP, Hutchinson K, Bramham J, McGowan A, Faul J, Cormican L (2017). Vitamin D Improves Selected Metabolic Parameters but Not Neuropsychological or Quality of Life Indices in OSA: A Pilot Study. J Clin Sleep Med JCSM Off Publ Am Acad Sleep Med.

[91] LeBlanc ES, Hedlin H, Qin F, Desai M, Wactawski-Wende J, Perrin N (2015). Calcium and vitamin D supplementation do not influence menopause-related symptoms: Results of the Women’s Health Initiative Trial. Maturitas.

[92] Nowak A, Boesch L, Andres E, Battegay E, Hornemann T, Schmid C (2016). Effect of vitamin D3 on self-perceived fatigue: A double-blind randomized placebo-controlled trial. Medicine (Baltimore).

[93] Roy S, Sherman A, Monari-Sparks MJ, Schweiker O, Hunter K (2014). Correction of Low Vitamin D Improves Fatigue: Effect of Correction of Low Vitamin D in Fatigue Study (EViDiF Study). North Am J Med Sci.

[94] Schöttker B, Kuznia S, Laetsch DC, Czock D, Kopp-Schneider A, Caspari R (2020). Protocol of the VICTORIA study: personalized vitamin D supplementation for reducing or preventing fatigue and enhancing quality of life of patients with colorectal tumor - randomized intervention trial. BMC Cancer.

[95] Havdahl A, Mitchell R, Paternoster L, Davey Smith G. Investigating causality in the association between vitamin D status and self-reported tiredness. Sci Rep. 2019;9. 10.1038/s41598-019-39359-z PMC6393455 30814568

[96] Hollen PJ, Msaouel P, Gralla RJ (2015). Determining issues of importance for the evaluation of quality of life and patient-reported outcomes in breast cancer: results of a survey of 1072 patients. Breast Cancer Res Treat.

