# Supplementary material for: Serum 25-hydroxyvitamin D and cancer-related fatigue: associations and effects on depression, anxiety, functional capacity and health-related quality of Life in breast cancer survivors during adjuvant endocrine therapy
Source: BMC Cancer. 2022 Aug 6;22:860. doi: 10.1186/s12885-022-09962-x (PMC9357315; doi:10.1186/s12885-022-09962-x)
Supplement: Supplementary file 1 — Additional file 1: Supplementary Table 1. Demographic and clinical characteristics of the breast cancer survivors during endocrine therapy, considering FACIT-Fatigue subgroups. [file 12885_2022_9962_MOESM1_ESM.docx]

**Supplementary Table 1.** Demographic and clinical characteristics of the breast cancer survivors during endocrine therapy, considering FACIT-Fatigue subgroups.

| Characteristics | FACIT-Fatigue | | *p* |
| --- | --- | --- | --- |
|  | Score < 34  (n = 32) | Score ≥ 34  (n = 57) |  |
| Age (years) |  |  |  |
| < 60 | 12 (48.0) | 13 (52.0) | 0.139ᶿ |
| ≥ 60 | 20 (31.3) | 44 (68.8) |  |
| Marital Status |  |  |  |
| Single/ Divorced/Separated/Widow | 18 (36.0) | 32 (64.0) | 0.992ᶿ |
| Married | 14 (35.9) | 25 (64.1) |  |
| Partner |  |  |  |
| No | 9 (40.9) | 13 (59.1) | 0.577ᶿ |
| Yes | 23 (34.3) | 44 (65.7) |  |
| Educational Level |  |  |  |
| Below high school | 23 (37.7) | 38 (62.3) | 0.612ᶿ |
| High school or higher education | 9 (32.1) | 19 (67.9) |  |
| Income (minimum wage) |  |  |  |
| < 3 | 23 (43.4) | 30 (56.6) | 0.076ᶿ |
| ≥ 3 | 27 (75.0) | 9 (25.0) |  |
| Work activity |  |  |  |
| Active | 10 (45.5) | 12 (54.5) | 0.285ᶿ |
| Inactive | 22 (32.8) | 45 (67.2) |  |
| Surgery |  |  |  |
| Breast-conserving surgery | 25 (49.0) ^a^ | 26 (51.0) ^b^ | 0.003ᶿ |
| Mastectomy | 7 (18.4) ^a^ | 31 (81.6) ^b^ |  |
| Prior Radiotherapy |  |  |  |
| No | 1 (7.1) ^a^ | 13 (92.9) ^b^ | 0.014ᶿ |
| Yes | 31 (41.3) ^a^ | 44 (58.7) ^b^ |  |
| Prior Chemotherapy |  |  |  |
| No | 4 (19.0) | 17 (81.0) | 0.065ᶿ |
| Yes | 28 (41.2) | 40 (58.8) |  |
| Chemotherapy Regimen |  |  |  |
| Adjuvant | 22 (41.5) | 31 (58.5) | 0.916ᶿ |
| Neoadjuvant | 6 (40.0) | 9 (60.0) |  |
| Priror Tamoxifen |  |  |  |
| No | 17 (34.7) | 32 (65.3) | 0.784ᶿ |
| Yes | 15 (37.5) | 25 (62.5) |  |
| Tumoral Subtype |  |  |  |
| Ductal | 32 (37.2) | 54 (62.8) | 0.550ᶷ |
| Lobular | 0 (0.0) | 3 (100.0) |  |
| Clinical Stage |  |  |  |
| I | 8 (30.8) | 18 (69.2) |  |
| II | 19 (39.6) | 29 (60.4) | 0.761ᶷ |
| III | 5 (38.5) | 8 (61.5) |  |
| NR | 0 (0) | 2 (100.0) |  |
| Tumor Grade |  |  |  |
| G1 | 3 (21.4) | 11 (78.6) |  |
| G2 | 24 (36.4) | 42 (63.6) | 0.121ᶷ |
| G3 | 4 (80.0) | 1 (20.0) |  |
| NR | 1 (25.0) | 3 (75.0) |  |
| Molecular Subtype |  |  |  |
| ER+ and/or PR+, HER2- and Ki-67<14% | 11 (37.9) | 18 (62.1) |  |
| ER+ and/or PR+, HER2- and Ki-67≥14% | 17 (45.9) | 20 (54.1) | 0.178ᶷ |
| ER+ and/or PR+, HER2+ | 3 (17.6) | 14 (82.4) |  |
| NR | 1 (16.7) | 5 (83.3) |  |
| Months since start on AI | 23.3 (16.6 – 36.9) | 33.6 (18.6 -49.2) | 0.028* |
| Years since diagnosis | 3 (2 – 4.8) | 4 (2.5 – 5) | 0.137* |
| Years since last menstrual period | 16.5 (6 – 19.8) | 15 (8 – 20) | 0.584* |

Continuous variables are shown as median (p25-p75), and categorical variables are shown as absolute numbers and percentage frequency (in parentheses); Time point: T0, Baseline; FACIT-Fatigue, Functional Assessment of Chronic Illness Therapy - Fatigue Scale, with cut-off < 34 indicating cancer-related fatigue; Prior, before starting AI use; AI, aromatase inhibitor; ER, estrogen receptor; PR, progesterone receptor; HER2, human epidermal growth factor type 2 receptor; Ki 67, Ki 67 antigen; -, negative; +, positive; NR: Not reported; G1: Well-differentiated tumor (low grade); G2: Moderately differentiated tumor (intermediate grade); G3: Poorly differentiated tumor (high grade). The Brazilian minimum wage was R$ 880.00. ᶿChi-Square Independence Test; ᶷFisher Exact Test; *Mann-Whitney. Different superscript letters represent statistical significance when comparing column proportions. Bold value is statistically significant at p < 0.05.
