# Supplementary material for: Serum 25-hydroxyvitamin D and cancer-related fatigue: associations and effects on depression, anxiety, functional capacity and health-related quality of Life in breast cancer survivors during adjuvant endocrine therapy
Source: BMC Cancer. 2022 Aug 6;22:860. doi: 10.1186/s12885-022-09962-x (PMC9357315; doi:10.1186/s12885-022-09962-x)
Supplement: Supplementary file 2 — Additional file 2: Supplementary Table 2. Direct and reverse association between 25(OH)D and cancer-related fatigue. [file 12885_2022_9962_MOESM2_ESM.docx]

**Supplementary Table 2.** Direct and reverse association between 25(OH)D and cancer-related fatigue.

| **FACIT-F Scale**  **(Mean ± SD)** | **Baseline (n = 89)** | | | | | | | **Longitudinal phase^1^ (n = 38)** | | | | | | |
| --- | --- | --- | --- | --- | --- | --- | --- | --- | --- | --- | --- | --- | --- | --- |
|  | **25(OH)D** | | **β** | **95% CI** | | **Wald Chi-square** | ****p*** | **Time points** | | **25(OH)D** | | **Model Effects Test** | | |
|  | **< 75nmol/L** | **≥ 75nmol/L** |  | **Lower** | **Upper** |  |  |  |  | **< 75nmol/L** | ≥ 75nmol/L | **Effects** | **Df** | *****p*** |
| **Model 1** | 36.47 ± 1.64 | 36.64 ± 1.32 | -0.173 | -4.315 | 3.969 | 0.007 | 0.935 |  |  | 36.47 ± 1.80 | 38.70 ± 1.47 |  |  |  |
|  |  |  |  |  |  |  |  | **T0** | 35.86 ± 1.60 | 35.33 ± 2.25 | 36.39 ± 1.72 | **Time points** | 2 | 0.065 |
|  |  |  |  |  |  |  |  | **T1** | 37.66 ± 1.76 | 36.40 ± 2.66 | 38.91 ± 1.71 | **25(OH)D** | 1 | 0.196 |
|  |  |  |  |  |  |  |  | **T2** | 39.24 ± 1.57 | 37.68 ± 2.02 | 40.79 ± 1.83 | **Interaction^2^** | 2 | 0.769 |
| **Model 2** | 35.34 ± 2.34 | 34.34 ± 2.06 | 1.007 | -3.327 | 5.342 | 0.207 | 0.649 |  |  | 38.43 ± 2.02 | 40.23 ± 1.68 |  |  |  |
|  |  |  |  |  |  |  |  | **T0** | 37.60 ± 1.82 | 37.35 ± 2.43 | 37.85 ± 1.93 | **Time points** | 2 | 0.062 |
|  |  |  |  |  |  |  |  | **T1** | 39.40 ± 1.95 | 38.35 ± 2.83 | 40.44 ± 1.89 | **25(OH)D** | 1 | 0.308 |
|  |  |  |  |  |  |  |  | **T2** | 40.99 ± 1.77 | 39.60 ± 2.20 | 42.39 ± 1.99 | **Interaction^2^** | 2 | 0.721 |
| **Model 3** | 36.16 ± 2.52 | 34.96 ± 2.34 | 1.203 | -3.187 | 5.594 | 0.289 | 0.591 |  |  | 38.60 ± 2.15 | 40.29 ± 1.80 |  |  |  |
|  |  |  |  |  |  |  |  | **T0** | 37.75 ± 2.00 | 37.55 ± 2.57 | 37.95 ± 2.13 | **Time points** | 2 | 0.097 |
|  |  |  |  |  |  |  |  | **T1** | 39.52 ± 2.06 | 38.62 ± 3.04 | 40.42 ± 1.96 | **25(OH)D** | 1 | 0.353 |
|  |  |  |  |  |  |  |  | **T2** | 41.07 ± 1.86 | 39.64 ± 2.29 | 42.51 ± 2.13 | **Interaction^2^** | 2 | 0.716 |
| **Model 4** | 36.34 ± 2.33 | 34.25 ± 2.17 | 2.090 | -1.992 | 6.172 | 1.007 | 0.316 |  |  | 38.62 ± 2.17 | 40.28 ± 1.82 |  |  |  |
|  |  |  |  |  |  |  |  | **T0** | 37.75 ± 2.02 | 37.56 ± 2.59 | 37.94 ± 2.16 | **Time points** | 2 | 0.099 |
|  |  |  |  |  |  |  |  | **T1** | 39.52 ± 2.08 | 38.63 ± 3.05 | 40.42 ± 1.98 | **25(OH)D** | 1 | 0.364 |
|  |  |  |  |  |  |  |  | **T2** | 41.06 ± 1.89 | 39.66 ± 2.31 | 42.47 ± 2.15 | **Interaction^2^** | 2 | 0.721 |
| **Model 5** | 36.22 ± 2.27 | 33.99 ± 2.12 | 2.224 | -1.761 | 6.209 | 1.196 | 0.274 |  |  | 38.61 ± 2.18 | 40.27 ± 1.83 |  |  |  |
|  |  |  |  |  |  |  |  | **T0** | 37.79 ± 2.03 | 37.67 ± 2.62 | 37.90 ± 2.17 | **Time points** | 2 | 0.108 |
|  |  |  |  |  |  |  |  | **T1** | 39.49 ± 2.10 | 38.53 ± 3.08 | 40.45 ± 1.99 | **25(OH)D** | 1 | 0.368 |
|  |  |  |  |  |  |  |  | **T2** | 41.05 ± 1.90 | 39.63 ± 2.32 | 42.46 ± 2.16 | **Interaction^2^** | 2 | 0.689 |
| **Model 6** | 36.67 ± 2.26 | 34.11 ± 2.10 | 2.554 | -1.400 | 6.507 | 1.603 | 0.205 |  |  | 38.59 ± 2.20 | 40.29 ± 1.86 |  |  |  |
|  |  |  |  |  |  |  |  | **T0** | 37.65 ± 2.69 | 37.52 ± 3.18 | 37.78 ± 2.77 | **Time points** | 2 | 0.669 |
|  |  |  |  |  |  |  |  | **T1** | 39.48 ± 2.12 | 38.49 ± 3.11 | 40.47 ± 2.02 | **25(OH)D** | 1 | 0.361 |
|  |  |  |  |  |  |  |  | **T2** | 41.19 ± 2.70 | 39.75 ± 2.92 | 42.62 ± 2.99 | **Interaction^2^** | 2 | 0.691 |
| **25(OH)D**  **(Mean ± SD)** | **Baseline (n = 89)** | | | | | | | **Longitudinal phase^3^ (n = 38)** | | | | | | |
|  | **FACIT-Fatigue** | | **β** | **95% CI** | | **Wald Chi-square** | ****p*** | **Time points** | | **FACIT-Fatigue** | | **Model Effects Test** | | |
|  | Score < 34 | Score ≥ 34 |  | **Lower** | **Upper** |  |  |  |  | **Score < 34** | **Score ≥ 34** | **Effects** | **Df** | *****p*** |
| **Model 1** | 30.43 ± 1.72 | 33.06 ± 1.29 | 2.631 | -1.616 | 6.877 | 1.474 | 0.225 |  |  | 32.33 ± 1.66 | 32.84 ± 1.15 |  |  |  |
|  |  |  |  |  |  |  |  | **T0** | 33.69 ± 1.27^a,b^ | 34.06 ± 1.78 | 33.33 ± 1.59 | **Time points** | 2 | **0.045** |
|  |  |  |  |  |  |  |  | **T1** | 34.08 ± 1.45^a^ | 34.41 ± 2.27 | 33.75 ± 1.46 | **FACIT-Fatigue** | 1 | 0.772 |
|  |  |  |  |  |  |  |  | **T2** | 29.98 ± 1.62^b^ | 28.51 ± 2.80 | 31.45 ± 1.39 | **Interaction^4^** | 2 | 0.513 |
| **Model 2** | 30.02 ± 2.25 | 31.34 ± 2.04 | 1.319 | -2.869 | 5.507 | 0.381 | 0.537 |  |  | 33.40 ± 1.99 | 33.15 ± 1.31 |  |  |  |
|  |  |  |  |  |  |  |  | **T0** | 34.22 ± 1.49 | 34.87 ± 2.00 | 33.57 ± 1.74 | **Time points** | 2 | 0.060 |
|  |  |  |  |  |  |  |  | **T1** | 34.78 ± 1.67 | 35.50 ± 2.49 | 34.05 ± 1.59 | **FACIT-Fatigue** | 1 | 0.890 |
|  |  |  |  |  |  |  |  | **T2** | 30.83 ± 1.88 | 29.84 ± 3.11 | 31.82 ± 1.52 | **Interaction^4^** | 2 | 0.572 |
| **Model 3** | 29.00 ± 2.52 | 30.50 ± 2.25 | 1.504 | -2.708 | 5.717 | 0.490 | 0.484 |  |  | 31.67 ± 2.91 | 31.58 ± 2.25 |  |  |  |
|  |  |  |  |  |  |  |  | **T0** | 32.26 ± 2.56^a,b^ | 32.86 ± 3.00 | 31.66 ± 2.60 | **Time points** | 2 | 0.060 |
|  |  |  |  |  |  |  |  | **T1** | 33.27 ± 2.50^a^ | 33.82 ± 3.19 | 32.71 ± 2.34 | **FACIT-Fatigue** | 1 | 0.961 |
|  |  |  |  |  |  |  |  | **T2** | 29.34 ± 2.72^b^ | 28.33 ± 3.74 | 30.35 ± 2.39 | **Interaction^4^** | 2 | 0.598 |
| **Model 4** | 29.45 ± 2.52 | 30.06 ± 2.26 | 0.611 | -3.796 | 5.018 | 0.074 | 0.786 |  |  | 31.99 ± 2.93 | 31.80 ± 2.26 |  |  |  |
|  |  |  |  |  |  |  |  | **T0** | 32.55 ± 2.58 | 33.23 ± 3.02 | 31.87 ± 2.60 | **Time points** | 2 | 0.056 |
|  |  |  |  |  |  |  |  | **T1** | 33.56 ± 2.51 | 34.19 ± 3.22 | 32.94 ± 2.35 | **FACIT-Fatigue** | 1 | 0.922 |
|  |  |  |  |  |  |  |  | **T2** | 29.57 ± 2.73 | 28.55 ± 3.74 | 30.59 ± 2.40 | **Interaction^4^** | 2 | 0.568 |
| **Model 5** | 29.45 ± 2.52 | 30.10 ± 2.26 | 0.651 | -3.767 | 5.069 | 0.083 | 0.773 |  |  | 31.97 ± 2.93 | 31.81 ± 2.25 |  |  |  |
|  |  |  |  |  |  |  |  | **T0** | 32.52 ± 2.57 | 33.14 ± 3.03 | 31.90 ± 2.60 | **Time points** | 2 | 0.059 |
|  |  |  |  |  |  |  |  | **T1** | 33.56 ± 2.51 | 34.20 ± 3.22 | 32.92 ± 2.34 | **FACIT-Fatigue** | 1 | 0.934 |
|  |  |  |  |  |  |  |  | **T2** | 29.59 ± 2.73 | 28.57 ± 3.75 | 30.60 ± 2.39 | **Interaction^4^** | 2 | 0.581 |
| **Model 6** | 29.87 ± 2.56 | 30.10 ± 2.25 | 0.229 | -4.272 | 4.729 | 0.010 | 0.921 |  |  | 31.83 ± 2.95 | 31.71 ± 2.28 |  |  |  |
|  |  |  |  |  |  |  |  | **T0** | 33.46 ± 2.85 | 34.05 ± 3.26 | 32.88 ± 2.89 | **Time points** | 2 | 0.058 |
|  |  |  |  |  |  |  |  | **T1** | 33.44 ± 2.54 | 34.05 ± 3.25 | 32.83 ± 2.37 | **FACIT-Fatigue** | 1 | 0.950 |
|  |  |  |  |  |  |  |  | **T2** | 28.40 ± 3.12 | 27.38 ± 4.05 | 29.42 ± 2.83 | **Interaction^4^** | 2 | 0.592 |

*Generalized linear models (GLzM); **General Mixed Model (GMM); Data adjusted for age, education level, income, body mass index, physical exercise and usage time of aromatase inhibitors. Time point: T0, Baseline; T1, Intermediate period, corresponding to 12 months after T0; and T2, Final follow-up period, corresponding to 24 months after T0; PRO Instrument, Patient-Reported Outcome Instrument; 25(OH)D, 25-hydroxyvitamin D; FACIT-Fatigue, Functional Assessment of Chronic Illness Therapy - Fatigue Scale, with cut-off < 34 indicating cancer-related fatigue; SD, Standard deviation; Df, degrees of freedom. Significant Model Effects Test are in bold. Sidak test: Different superscript letters represent statistical significance when comparing pairs, p value <0.05. ^1^Longitudinal phase: FACIT-Fatigue score is shown as mean ± SD; ^2^Interaction between 25(OH)D and time points of study; ^3^Longitudinal phase: 25(OH)D concentration is shown as mean ± SD; ^4^Interaction between FACIT-Fatigue and time points of study.

Model 1: Data adjusted for age

Model 2: Data adjusted for age and education level

Model 3: Data adjusted for age, education level and income

Model 4: Data adjusted for age, education level, income and body mass index

Model 5: Data adjusted for age, education level, income, body mass index and physical exercise

Model 6: Data adjusted for age, education level, income, body mass index, physical exercise and usage time of aromatase inhibitors
